# Global chromatin reorganization and regulation of genes with specific evolutionary ages during differentiation and cancer

**DOI:** 10.1093/nar/gkaf084

**Published:** 2025-02-18

**Authors:** Flavien Raynal, Kaustav Sengupta, Dariusz Plewczynski, Benoît Aliaga, Vera Pancaldi

**Affiliations:** CRCT, Université de Toulouse, Inserm, CNRS, Université Toulouse III-Paul Sabatier, Centre de Recherches en Cancérologie de Toulouse, 31100 Toulouse, France; Laboratory of Functional and Structural Genomics, Center of New Technologies (CeNT), University of Warsaw, Mazowieckie, 02-097 Warsaw, Poland; Faculty of Mathematics and Information Science, Warsaw University of Technology, 00-662 Warsaw, Poland; Department of Molecular Genetics, Erasmus University Medical Center, Erasmus MC Cancer Institute, 3015 GD Rotterdam, the Netherlands; Laboratory of Functional and Structural Genomics, Center of New Technologies (CeNT), University of Warsaw, Mazowieckie, 02-097 Warsaw, Poland; Faculty of Mathematics and Information Science, Warsaw University of Technology, 00-662 Warsaw, Poland; CRCT, Université de Toulouse, Inserm, CNRS, Université Toulouse III-Paul Sabatier, Centre de Recherches en Cancérologie de Toulouse, 31100 Toulouse, France; CRCT, Université de Toulouse, Inserm, CNRS, Université Toulouse III-Paul Sabatier, Centre de Recherches en Cancérologie de Toulouse, 31100 Toulouse, France; Barcelona Supercomputing Center, 08034 Barcelona, Spain

## Abstract

Cancer cells are highly plastic, favoring adaptation to changing conditions. Genes related to basic cellular processes evolved in ancient species, while more specialized genes appeared later with multicellularity (metazoan genes) or even after mammals evolved. Transcriptomic analyses have shown that ancient genes are up-regulated in cancer, while metazoan-origin genes are inactivated. Despite the importance of these observations, the underlying mechanisms remain unexplored. Here, we study local and global epigenomic mechanisms that may regulate genes from specific evolutionary periods. Using evolutionary gene age data, we characterize the epigenomic landscape, gene expression regulation, and chromatin organization in several cell types: human embryonic stem cells, normal primary B-cells, primary chronic lymphocytic leukemia malignant B-cells, and primary colorectal cancer samples. We identify topological changes in chromatin organization during differentiation observing patterns in Polycomb repression and RNA polymerase II pausing, which are reversed during oncogenesis. Beyond the non-random organization of genes and chromatin features in the 3D epigenome, we suggest that these patterns lead to preferential interactions among ancient, intermediate, and recent genes, mediated by RNA polymerase II, Polycomb, and the lamina, respectively. Our findings shed light on gene regulation according to evolutionary age and suggest this organization changes across differentiation and oncogenesis.

## Graphical Abstract

**Graphical Abstract:**
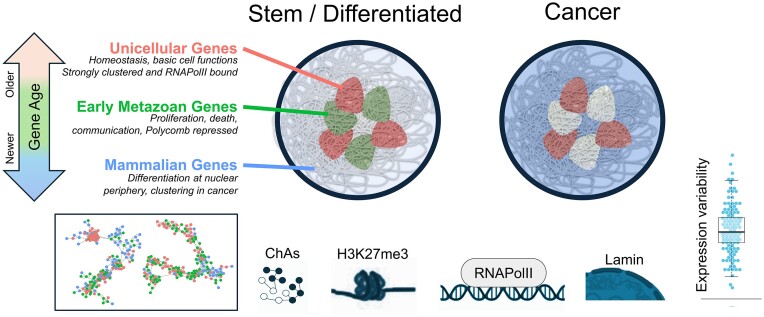

## Introduction

Despite recent progress, our understanding of oncogenesis remains incomplete. The loss of cellular identity is a complex and multi-step process, and is characterized by processes at different levels (genetic, epigenetic, etc.). In the last decades, two major theories about oncogenesis were proposed: the oncogenes paradigm, which views cancer primarily as a genetic disease, and epigenetic oncogenesis [1]. Evolutionary biologists also hypothesize that cancer is the result of a breakdown of multicellular cooperation [2], consistent with the fact that most cancer genes date back to unicellular organisms and the transition to multicellularity and complex body plans [3–5].

The ∼20 000 human coding genes emerged at different evolutionary ages and evolved over time, notably with DNA mutations and duplication events. Gene and epigenome evolved differentially. Indeed, a subset of chromatin marks, like Polycomb deposited H3K27me3 and promoter-associated H3K4me3, were conserved more often than expected at orthologous regions with low substitution rates. Three chromatin marks (DNA methylation, H3K36me3, and H3K27ac) in mammal ESCs are independent of DNA sequence evolution [6]. With the genomic phylostratigraphy method, genes can be dated by looking at their homologs across species [7]. Other methods have been proposed in the literature to estimate evolutionary gene age, such as studying duplicated genes, as demonstrated by Juan *et al.* [8]. In this paper the authors highlight a correspondence between gene age, replication timing, expression breadth across tissues, and their propensity to accumulate copy number alterations. They suggest a scenario in which old housekeeping genes are somehow protected from variations (both transcriptional and sequence-based), while younger genes are located in regions of high variability, promoting their evolvability and restricting the impacts of their evolution to specific tissues [8]. The spatial organization of genes within the nucleus is also thought to influence the segregation of genes of different ages [9].

In 2017, Trigos *et al.* showed that the gene expression of evolutionary old genes increased in cancer, without explaining the underlying mechanism [10–12]. Transcriptomics analyses of seven solid tumors showed a significant global increase in the expression of genes originating from unicellular organisms, while genes of metazoan origin were inactivated in cancer [12]. The widespread expression of unicellular genes in cancer appears to be a general pattern, but the specific up-regulation of these genes varies according to their function, indicating that this process is regulated rather than random [12, 13]. More interestingly, cancer cells show expression of genes that are specific to embryogenesis and are not expressed in healthy adult tissues [14].

These findings raised new interest in a theory that implicated the reactivation of unicellular programs in cancer, namely the atavistic theory from 1914 [15], which saw the malignant tumor cell as a previously normal and “altruistic” tissue cell that changed into an “egotistical” mode with a loss of its specific functions. Recently, purely epigenomic mechanisms, namely the transient inactivation of Polycomb, were shown to be sufficient for the initiation of cancer in the fruit fly [16], while embryonal programs are often implicated in dedifferentiation in cancer [17–19], and 3D chromatin rearrangements are well-known hallmarks of cancer as they unlock phenotypic plasticity [20, 21].

Despite findings on the relationships between gene ages, expression levels, and their regulation across oncogenesis, the underlying mechanisms are unclear. Both the local chromatin context and 3D organization of the genome within the nucleus have been shown to be cell type specific and to be altered during differentiation and oncogenesis, suggesting their potential role.

Recent work has shown that promoter DNA sequence characteristics, including general GC content, TATA-box motifs, and number of transcription factor (TF) binding motifs, can be predictive of gene expression variability (EV) across tissues, across individuals, and across single cells in multiple species [22, 23], suggesting that the local chromatin environment, as well as sequence characteristics, can impact expression regulation and variability.

On the other hand, the global chromatin context of genes is likely to also play a role. The 2-m-long chain of DNA folds into 3D space and coils inside the nucleus of each cell, giving rise to several hierarchical structures, which can be different in each cell but can also globally reflect cell type and cell state [24, 25]. Despite consistent progress in our understanding of chromatin organization, there are still open questions about how the characteristics of the 3D epigenome can be related to cellular processes and phenotypes [26–28]. To investigate this, a useful framework involves representing chromatin as a network in which nodes are chromatin fragments and edges are experimentally detected spatial contacts between fragments [29].

Here, we investigate potential local and global epigenomic mechanisms that could mediate the regulation of genes of specific evolutionary ages. We consider human embryonic stem cells (hESCs), several differentiated immune cells, leukemic cells, and cells from different stages of colorectal cancer (CRC) to study the gene evolutionary classes’ epigenomic characteristics, their spatial patterns inside the nucleus, and their regulation across differentiation and oncogenesis. Integrating chromatin 3D structure data with epigenomic features using network approaches, we propose that different mechanisms could be responsible for the physical organization of genes of specific evolutionary ages. Specifically, ancient genes of unicellular origins and with housekeeping functions are mostly regulated and clustered by RNA polymerase 2, intermediate metazoan genes, often involved in oncogenesis, are mostly coordinately repressed and clustered by Polycomb factors and, finally, novel mammalian specific genes, often found to be more peripheral in the nucleus, are bound and coordinately repressed by the lamina.

## Materials and methods

### Gene ages

We used the gene age dataset from Trigos *et al.*, which estimated gene ages through phylostratigraphy [12]. Here, 19.404 genes are classified into 16 age classes, from unicellular genes to Homo sapiens specific ones, which are grouped into three main gene age categories: unicellular ancestor (UC, 7.397 genes), early metazoan (EM, 8.682 genes), and mammal-specific (MM, 3.325 genes).

### Expression and DNA methylation variability

Expression and methylation variability in monocytes, neutrophils, and T-cells were computed from 125 healthy individuals, using a method that accounted for confounding effects due to the correlation between mean and variability measurements [30, 31]. To minimize the influence of genetic variability, Ecker *et al.* applied stringent quality control, corrected batch effects with ComBat, ensuring high consistency between cross-over samples (*r* = 0.96), and performed paired analyses within individuals to reduce inter-individual differences. The analysis focused on protein-coding genes expressed in ≥50% of individuals within each cell type, prioritizing variability driven by regulatory and environmental factors rather than genetic background [31]. Fisher’s exact tests were performed using the geneOverlap (v1.32.0) R package [32].

### ChIP-seq processing

Histone modifications ChIP-seq datasets in monocytes were downloaded from the ENCODE portal (https://www.encodeproject.org/) [33] in BigWig format, with the ENCODE identifiers listed in Supplementary Table S1. Other ChIP-seq datasets were downloaded in FASTQ raw format. Sequenced reads were mapped to the human reference genome (built hg19/GRCh37) using Burrows–Wheeler Aligner (bwa v0.7.17-r1188) [34]. Binary Alignment and Map (BAM) files were obtained, sorted, and indexed by using Samtools utilities (v1.15.1) [35]. ChIP-seq peak calling was performed with MACS2 (v.2.2.7.1) using the respective inputs [36]. H3K27me3 peaks were called with the –broad parameter on.

### ChIP-seq analysis

ChIP-seq profiles were generated using the seqplots R package (v1.23.3) [37]. Polycomb target genes were defined as genes with strict TSS localization, according to EnsDb.Hsapiens.v75 Ensembl hg19 gene annotations, that overlap with an H3K27me3 peak. CpG localizations were analyzed using the ChIPSeeker R package (v1.32.1) [38].

### RNA-seq analysis

The raw read count matrix for hESC and hESC-derived cardiomyocytes was downloaded from GEO with accession number GSE69618 [39]. Differentially expressed genes were found using the DESeq2 R package (v1.36.0), filtering for log2 fold change (log2FC) >2 or ←2 and adjusted *P*-value < .1. Gene Ontology enrichment analysis was conducted using the limma (v3.52.1) [40] and clusterProfiler (v4.4.3) R packages [41].

### Pol II pausing index

We downloaded raw RNA polymerase II (Pol II) ChIP-seq FASTQ files for hESC (H9/WA09 cell line), B-cell (GM12878 cell line), and lymphoma B-cell (LY1 cell line) and processed them as described above. Pol II BigWig density files were generated using the bamCoverage tool from deepTools suite (v3.0.2) and normalized with the –normalizeUsing RPKM flag. To compute Pol II pausing indices, we defined the promoter region for each gene as TSS − 30 bp to TSS + 300 bp and the gene body region as TSS + 300 bp to TES, based on EnsDb.Hsapiens.v75 gene annotation. Pol II scores for overlapping bins within each region were averaged separately. The pausing index (PI) was calculated as the ratio of Pol II density in the promoter region to the density in the gene body region. Outliers in each cell’s PI distribution were detected and removed using the interquartile range method. Genes with a sum of Pol II pausing indices across the three cell types <0.2 were considered unexpressed and were excluded. Additionally, genes <1 kb, according to EnsDb.Hsapiens.v75 gene annotation, were discarded. To facilitate comparison across cells, each distribution was normalized to fall between 0 and 1.

### LAD-associated genes

Nuclear lamina-interacting genes were defined as genes with at least part of the gene body overlapping with lamina associated domains (LADs), according to EnsDb.Hsapiens.v75 Ensembl hg19 gene annotations and published “constitutive” and “facultative” LADs, either together or separately [42].

### PCHi-C chromatin networks and ChAs calculations

PChi-C datasets used with GEO accession numbers are summarized in Supplementary Table S1. Considering significant interactions with CHiCAGO scores >5, we constructed networks by considering DNA restriction fragments as nodes and interaction between fragments as edges. These networks were processed using the igraph R package (v1.3.2) [43]. Chromatin assortativity (ChAs) was calculated using the ChAseR R package (v0.0.0.9) (https://bitbucket.org/eraineri/ChAseR/) [44]. Gene age ChAs was computed using age classes in the categorical ChAs mode. For z-score calculations, we performed 100 randomizations preserving genomic distances (dist.match = TRUE). Chromatin networks were visualized using Cytoscape (v3.8.0) and Gephi (v0.10.1).

### ΔChAs calculation

We developed the ΔChAs method to identify chromatin features associated with the 3D clustering of specific gene groups (e.g. age categories) by determining whether these features significantly contribute to clustering a gene group together (ΔChAs > 0) or not (ΔChAs < 0). Within each category, two gene subgroups are created based on the presence or absence of a chromatin feature. ChAs z-scores for both subgroups are then calculated. Finally, ΔChAs is determined as the difference between the ChAs z-score of genes associated with the specific feature and the ChAs z-score of other genes.

## Results

### Gene expression and expression variability are strongly associated to evolutionary ages

To explore the interplay between genes’ evolutionary history, their regulation, and nuclear organization, we considered a dataset established through phylostratigraphy that assigns ages to genes based on their homologous relationships across species. The dataset comprises 19 404 human genes categorized into 16 evolutionary ages, which are further grouped into three principal age classes: unicellular (UC, 7.397 genes), early metazoan (EM, 8.682 genes), and mammal-specific (MM, 3.325 genes) (Supplementary Fig. S1A) [12]. Throughout the manuscript, we will primarily focus on the three major gene age groups for clarity and relevance in specific analyses.

We performed a functional enrichment analysis of genes within each category (Supplementary Fig. S1B), confirming that UC genes are enriched in fundamental cellular processes (catabolism, RNA splicing, histone modification) [5, 12]. This contrasts with later phylostrata genes, which are associated with more complex cellular functions (sensory perception, keratinization). Additionally, we explored the enrichment of housekeeping genes across all age classes (Supplementary Fig. S1C), observing a notable proportion of UC genes classified as housekeeping compared to EM and MM genes (18% of UC genes against 6% of EM genes and 1% of MM genes), while the EM class contains the majority of proto-oncogenes within the leukemic context [45], with some others being in the UC class (Supplementary Fig. S1D).

**Figure 1.**
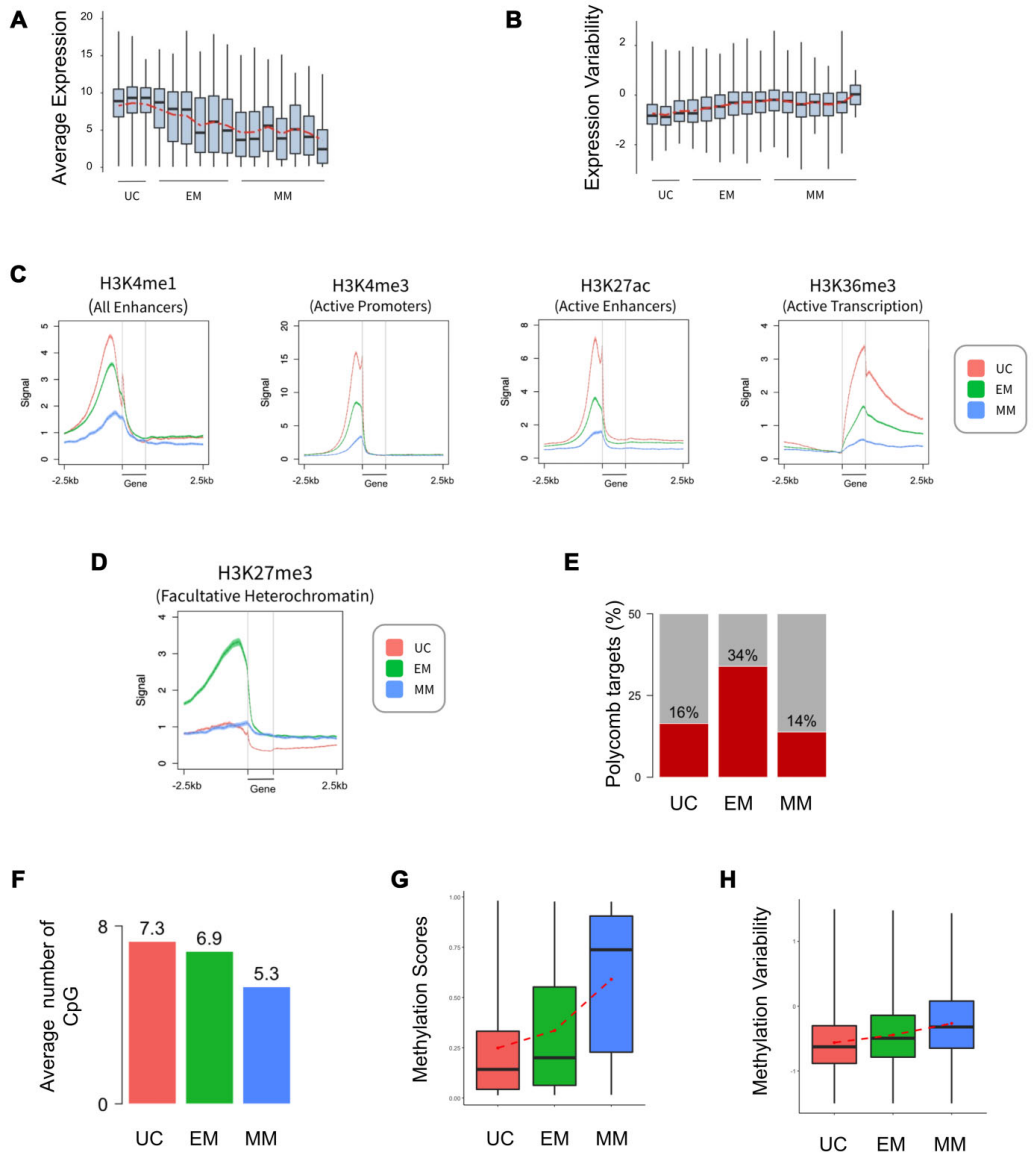
Genes of different evolutionary age classes display specific genomic and epigenomic features. (**A**) Boxplot of average inter-individual gene expression levels in monocytes across 16 gene age classes (Spearman Rho = −0.95, *P*-value < 2.2e-16). (**B**) Boxplot of inter-individual EV in monocytes across 16 gene age classes (Spearman Rho = 0.77, *P*-value = .00074). (**C**) Average epigenetic profiles around the three main gene age classes (UC unicellular genes, EM early metazoan genes, and MM mammal-specific genes) in monocytes. (**D**) Average H3K27me3 profile around the three main gene age classes (UC unicellular genes, EM metazoan genes, and MM mammal-specific genes) in monocytes. (**E**) Percentages of H3K27me3 repressed gene promoters in UC, EM, and MM ages in monocytes. (**F**) Average number of potential CpG islands by gene across the three main age categories in monocytes. (**G**) Average methylation scores computed by Chen *et al.* in monocytes across the three main age categories [48]. Methylation scores were assigned to the 440 905 detected methylated CpG islands and calculated as the ratio between the intensity of the methylated probe and the global intensity within each individual. (**H**) Average DNA methylation variability across the three main age categories in monocytes.

Healthy cells display abundant variability and plasticity in their phenotype, partly based on changes in the epigenome [31, 46], while EV can be related to cancer aggressiveness [47]. We therefore proceeded to investigate the relationship between gene age, mean expression, and EV. Across 125 healthy individuals [48] (Fig. 1A and Supplementary Fig. S1E), our analysis revealed an inverse correlation between the 16 gene ages and average expression levels across these cell types (Spearman test, Rho = −0.95, *P*-value < 2.2e-16 in monocytes), indicating that older genes exhibit higher average expression levels, consistent with their involvement in fundamental cellular processes.

In the following, we define variability as variation of values across instances (across individuals or across single cells) and plasticity as variation in response to changes in external conditions. Briefly, expression variability (EV) [30, 31] estimates the excess or reduced variability compared to what would be expected for the corresponding mean value of the feature. Since understanding EV is important for comprehending the underlying gene regulation mechanisms [46], we examined the relationship between EV and age across immune cell types (Fig. 1B, Supplementary Fig. S1F and G), observing that older genes have lower variability compared to newer genes (Spearman test, Rho = 0.77, *P*-value = .0007 in monocytes). UC genes are significantly enriched in low variability groups (groups 1–6), while EM genes are enriched in high variability groups (groups 8–10) for each of the three immune cell types considered. These results were validated when considering gene subsets with different variability but similar expression (Supplementary Fig. S1H).

To further validate our findings, we conducted additional analyses using data from Sigalova *et al.* [22], which provides insights into variability across isogenic Drosophila lines and human tissues. Sigalova *et al.* identified an association between gene promoter features (sequence-based and epigenomic) in defining the level of gene expression variation. Analyzing these features related to variability in combination with the previously introduced age classes, we found correlations between gene age and active TSS (Spearman Rho = −0.37, *P*-value < 2.2e-16), the number of TF binding motifs in gene promoters (Spearman Rho = −0.34, *P*-value < 2.2e-16), the number of gene exons (Spearman Rho = −0.27, *P*-value < 2.2e-16), promoter size (Spearman Rho = −0.13, *P*-value < 2.2e-16), and GC content (Spearman Rho = −0.12, *P*-value < 2.2e-16) (Supplementary Fig. S1I).

Given the strong correlations observed among evolutionary ages, gene expression levels, and EV, we asked whether genes of specific age classes exhibit distinct epigenomic landscapes, which might imply different regulatory mechanisms (Fig. 1C). In monocytes, we found histone modifications associated with gene activation (H3K4me1/3, H2K27ac, and H3K36me3) to be highly enriched around UC genes, intermediate intensity around EM genes, and the lowest intensity in MM genes. These histone modification density levels are consistent with increasing expression levels for older genes, suggesting that the local epigenetic environment of the genes is also closely related to their evolutionary ages.

### Polycomb proteins target mainly metazoan genes

We then analyzed histone modifications associated to transcriptional repression (Fig. 1D and Supplementary Fig. S1J). Interestingly, in monocytes the H3K27me3 repressive mark shows enrichment around intermediate EM genes, while both older UC and younger MM genes exhibit no enrichment, suggesting a specific regulatory role by Polycomb complexes for this gene age category. Conversely, H3K9me3 mark shows no enrichment for any gene age class. This observation led us to focus on genes targeted by Polycomb proteins. We assessed the proportion of genes from each age class enriched with H3K27me3 peaks on the promoter in monocytes (Fig. 1E). Results reveal a higher proportion of EM genes (34%) targeted by Polycomb complexes whereas UC and MM genes exhibit a lower proportion of targeted genes (16% and 14%, respectively).

Taken together, these findings suggest that Polycomb protein complexes predominantly regulate genes that emerged during the metazoan period. This aligns with the role of these genes in establishing body plans and their regulated suppression in early phases of development.

### CpG islands and DNA methylation variability are associated to evolutionary ages

Next, we directed our focus towards DNA methylation as a key epigenetic feature. The dataset provided by Chen *et al.* contains methylation scores for each of the 440 905 detected methylated CpG sites across the entire human genome in monocytes, gathered from 200 healthy individuals via whole genome bisulfite sequencing [48]. We first investigated the genome regions associated with these methylated CpGs, which showed that half of all studied CpG islands are localized around gene promoters (52.52% within 3 kb of the TSS, 42% within 1 kb) (Supplementary Fig. S1K) and explored the correlation between gene ages and DNA methylation patterns. Methylated CpG islands are thought to repress gene expression when found within promoters [49]. Our analysis indicated a progressive decrease in the average number of CpG islands within gene promoter regions across evolutionary gene age classes (Fig. 1F). We then analyzed methylation scores computed by Chen *et al.* in monocyte gene promoters classified according to their respective ages [48]. Results show a gradual increase in methylation levels for newer evolutionary ages, suggesting a preferential regulatory role of DNA methylation for MM genes (Fig. 1G). Interestingly, inter-individual DNA methylation variability exhibited a roughly linear increase from older to newer gene age classes (Fig. 1H), suggesting a more stable DNA methylation profile for older genes and a greater variability around newer genes. This pattern may reflect an evolutionary process in which genes transition over time from being silenced in highly methylated regions for younger genes to more optimized and straightforward activation programs in older genes.

### Most genes deregulated through cell differentiation appeared recently in evolution

The above observations led us to focus on several gene expression regulatory mechanisms that could elucidate the organization and maintenance of age-related gene expression patterns. We investigated whether these patterns remain stable across global phenotypic shifts during cell differentiation and oncogenesis.

We studied three distinct and extensively studied cell states for comparison: hESCs (H9), representing an undifferentiated state, differentiated cells (CD19 + primary B-cells, monocytes, neutrophils, T-cells, and cardiomyocytes), and B-cells from chronic lymphocytic leukemia (B-CLL) patients, as a representation of a cancerous leukemic cell state [31, 39].

**Figure 2.**
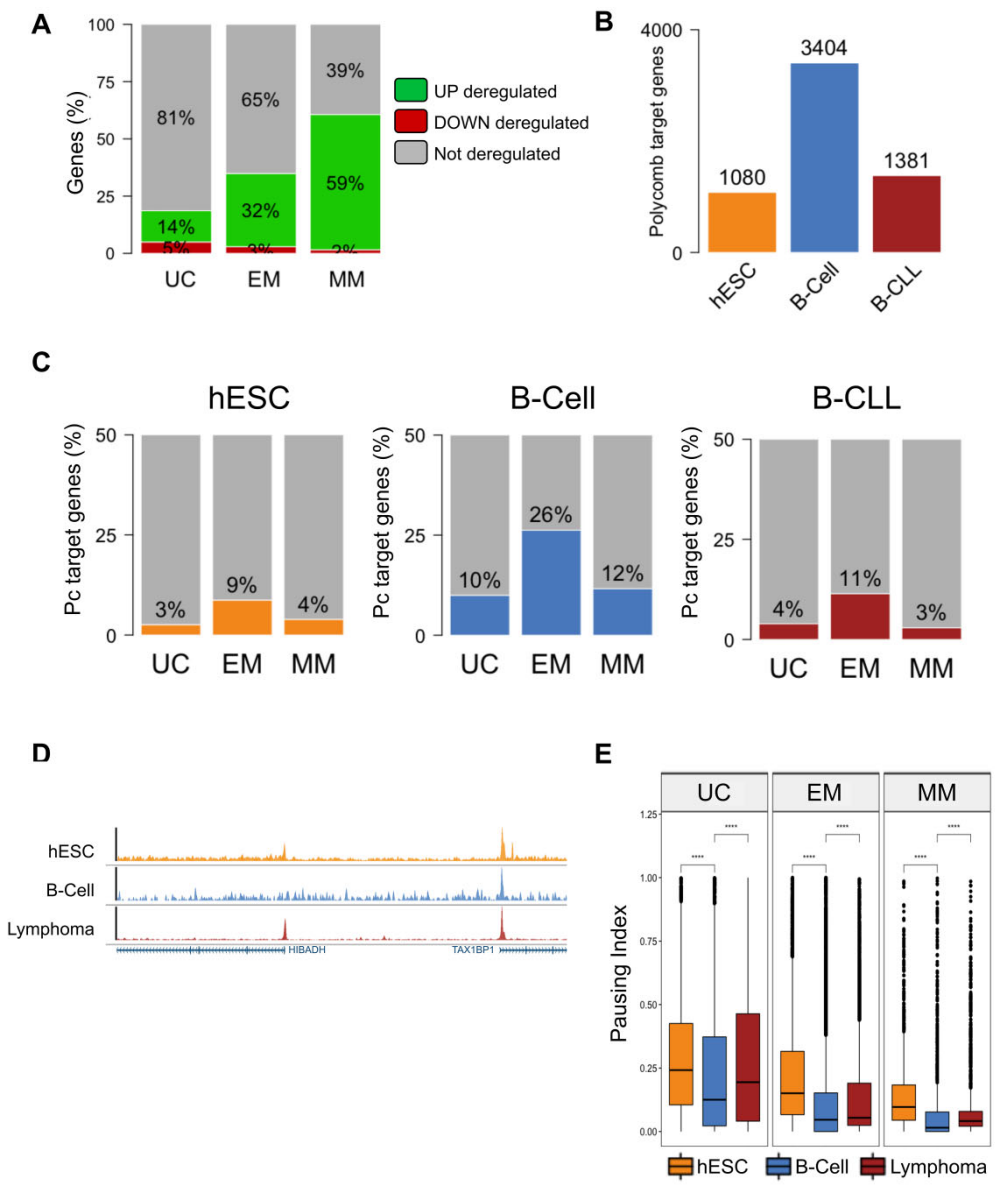
Gene expression regulation across different evolutionary ages is altered in differentiation and cancer. (**A**) Proportion of down-regulated, up-regulated, and non-deregulated genes in cardiomyocyte compared to hESC for the three main gene ages. (**B**) Number of Polycomb target genes in hESC, B-cell, and CLL. (**C**) Percentage of Polycomb target genes for the three main gene age categories in hESC (left), B-cell (middle), and CLL (right) cell states. (**D**) Browser snapshots of Pol II tracks in hESC, B-cell, and CLL. (**E**) Pol II PI distributions in hESC, B-cell, and CLL for each of the three main age groups. Statistical comparisons between groups were done by the Wilcoxon–Mann–Whitney test. n.s. = nonsignificant, ****P< .0001.

We first asked whether genes of specific evolutionary ages are the most regulated during cell differentiation and oncogenesis. We analyzed a gene expression dataset from hESC differentiation into cardiomyocytes [50] and computed proportions of up- and down-regulated genes within each evolutionary age class, observing a predominance of up-regulated genes across cell differentiation (from 5.382 differentially expressed genes, 89% are found up-regulated), which is consistent with acquisition of a lineage-specific gene expression profile (Fig. 2A; Supplementary Fig. S2A and B). Interestingly, this proportion of up-regulated genes rises across evolutionary age classes (14% of UC genes, 32% of EM genes, and 59% of MM genes). Conversely, a minority of genes exhibited down-regulation during cardiac differentiation, with the number of down-regulated genes being even lower for younger gene age classes (5% of UC genes, 3% of EM genes, and 2% of MM genes).

### Gene expression regulation of genes from different evolutionary ages is altered in differentiation and cancer

In order to study how gene expression regulation is affected during cell differentiation and oncogenesis, we first focused on Polycomb protein complexes. We assessed the presence of the H3K27me3 mark in hESC, B-cell, and CLL as a proxy for Polycomb binding, which is known to repress developmentally regulated genes and influence expression noise levels [51, 52].

The total count of Polycomb-targeted genes across the genome was computed for each cell state, revealing an increasing number of targeted genes during cell differentiation and a sharp decline through oncogenesis (Fig. 2B). Building upon our earlier findings indicating that EM genes were prominently regulated by Polycomb in monocyte (c.f. Fig. 1D and E), we observed a similar trend in stem cells, B-cells, and CLL cancer cells (Fig. 2C and Supplementary Fig. S2C), with the most notable impact of gene age class in B-cells compared to stem or cancer cells.

We then investigated gene expression regulation by RNA Pol II binding, another factor influencing transcription levels and variability, by analyzing Pausing RNA Pol II. This mechanism allows cells to modulate and synchronize transcription and can be quantified using the PI calculated as the ratio of Pol II signal density around gene TSS to the Pol II signal density along the gene body [53].

Pol II pausing indices were then computed for each gene in hESCs, B-cells, and B-CLL (Fig. 2D), and distributions were plotted for genes grouped by age class in each cell state, revealing a declining global distribution across evolutionary classes (Fig. 2E) and indicating that older genes are subject to tighter regulation by the Pol II pausing mechanism. Interestingly, we observed significant decreases in global Pol II pausing distributions during cell differentiation and significant increases during oncogenesis for every gene age class.

To interpret these results in the context of known cancer-related genes, we assessed the enrichment of COSMIC-classified oncogenes and tumor suppressor genes in the gene sets produced by each of the presented analyses. This revealed that a significant proportion of EM polycomb-targeted genes are oncogenes, whereas many UC genes with paused Pol II are tumor suppressors. (see Supplementary Fig. S2D–F and Supplementary Text S1 for description).

In summary, our findings indicate that the most recently evolved genes exhibit elevated expression levels during cell differentiation, while the oldest genes are most expressed during oncogenesis. EM genes are the primary targets of Polycomb complexes across all our cell states and are enriched in oncogenes. Notably, the percentage of genes targeted by Polycomb seems to increase in differentiated cells and subsequently decrease in cancer. Additionally, our analysis suggests that Pol II pausing is more pronounced in older genes, enriched in tumor suppressors, potentially influencing their expression regulation. With an opposite trend compared to Polycomb activity, this mechanism tends to decrease during cell differentiation and increase during carcinogenesis.

### The nuclear periphery is enriched with newer genes

Gene regulation, especially through Polycomb binding, has been strongly associated with the 3D chromatin structure within the nucleus, which was shown to be deeply compromised in Polycomb units mutants [54]. Chromatin organization is known to be dramatically reorganized both in cell differentiation through lineage definition [55], and in cancer [56, 57]. This prompted us to investigate whether gene evolutionary ages might also be spatially organized in the nucleus.

To explore the potential association between gene evolutionary ages and genome 3D structure, we investigated whether genes from specific age categories are more likely to be situated at the nucleus periphery. We obtained a dataset of computed LADs annotated by Kind *et al.* [42], across nine human cell lines, describing LADs as either facultative (cell type-specific) or constitutive (cell type-invariant).

**Figure 3.**
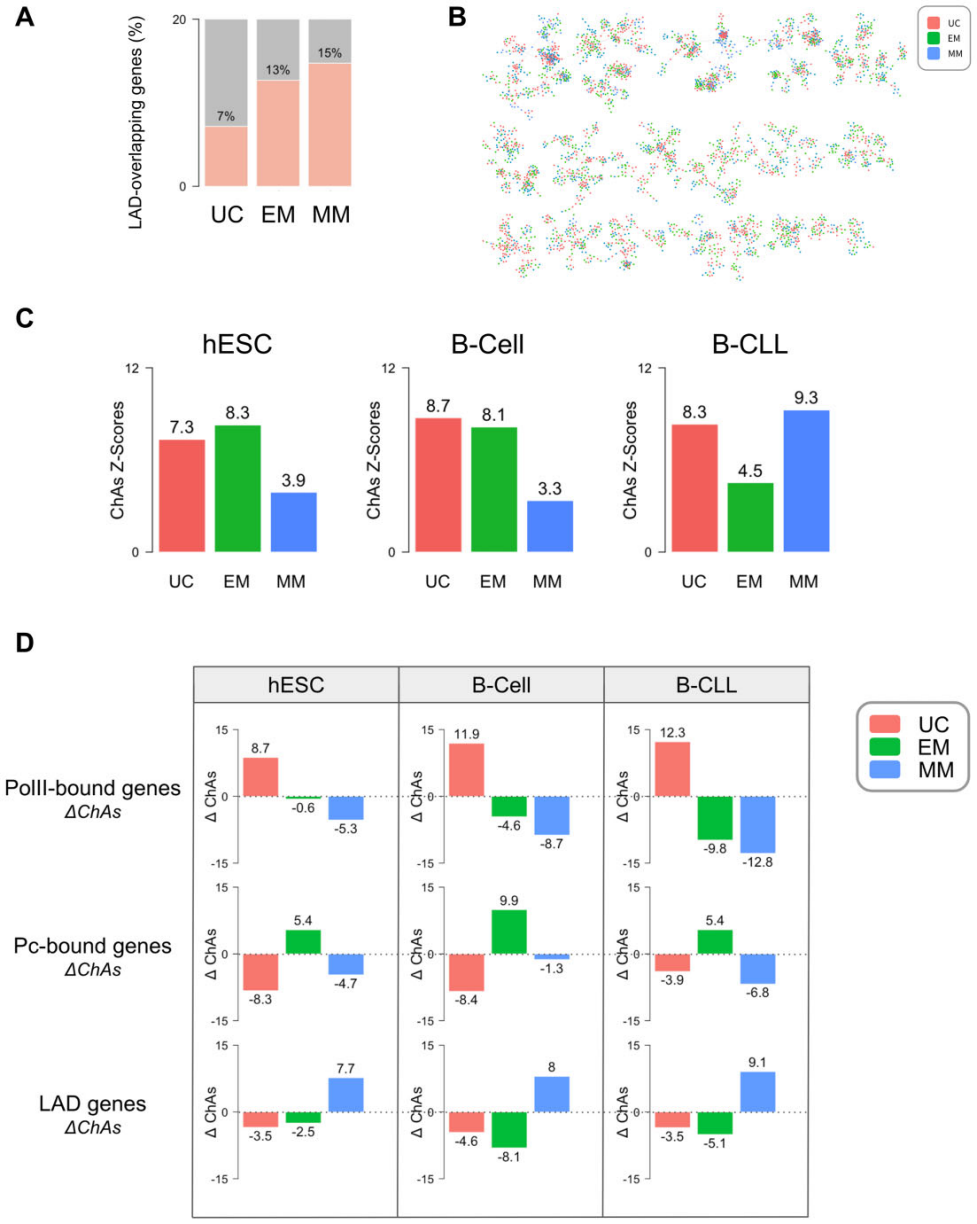
Spatial patterns of gene evolutionary age in 3D genome architecture. (**A**) Overlap with LADs annotated in multiple cell lines [42] for genes belonging to the different age classes. (**B**) Visualization of the 3D promoter–promoter interaction network in monocyte [59] with node color representing gene age classes. (**C**) Comparison of ChAs z-score of genes from different gene classes between hESCs, B-cells, and B-CLL. (**D**) ΔChAs z-scores of different chromatin features: Pol II (top), Polycomb (middle), and LAD (bottom) in hESC, B-cell, and B-CLL.

We considered genes contained in facultative and constitutive LADs either together or separately and examined their age. Interestingly, we observed a higher proportion of younger genes associated with LAD domains (15% and 13% of MM and EM age categories, respectively) compared to older genes (7% of UC genes), (Fig. 3A and Supplementary Fig. S3A). Our findings support that genes located at the nucleus periphery are predominantly of newer origin and are consistent with the lower expression levels observed for the youngest genes (c.f. Fig. 1A).

### Genes establish preferential contacts in the 3D chromatin network according to their age

In prior studies, we and others have demonstrated that numerous epigenomic features, including many TF binding peaks, gene expression, DNA methylation, histone modifications [44], replication timing, and replication origin activation [58], display strong clustering in 3D space. We used the same network framework and ChAs to identify which features cluster more significantly on the chromatin network than would be expected at random, considering solely linear proximity of the regions along the chromosome.

We exploited promoter-centered chromatin networks derived from PCHi-C datasets, where each node represents a DNA fragment and edges represent significant physical contacts (or proximity) between DNA fragments detected by the conformation capture protocol. We started by mapping gene ages on promoter only subnetworks in monocyte, neutrophil, and T-cell (Fig. 3B and Supplementary Fig. S3B) [59] and observed that genes from specific age classes tend to group together, forming age-specific clusters. We next confirmed this tendency using ChAs computation with the three broad gene age classes interpreted as separate binary categorical values (Supplementary Fig. S3C and D) [44]. We observed ChAs z-scores for every age category being significantly above 2 (equivalent to *P*-value = .05), indicating that contacts are more frequent than would be expected given the genomic proximity of the regions and the number of promoters in each class. Interestingly, UC genes emerge as the most assortative gene class in all three cell types, consistently followed by EM genes. Conversely, the most recent gene age category displays lower assortativity across the three immune cell types.

These findings suggest that in fully differentiated cells, the oldest unicellular genes exhibit stronger connectivity among themselves, forming prominent clusters in the chromatin network, closely trailed by EM genes, which also demonstrate preferential interactions. The newest genes have less pronounced preferential interactions, with ChAs z-score values just above significance.

Having observed specific interactions among genes of same evolutionary ages in monocyte, neutrophil, and T-cell, we next investigated the potential alteration of this age-related 3D conformational arrangement during cell differentiation and oncogenesis. We calculated ChAs z-scores on PCHi-C promoter subnetworks from hESC, normal B-cells, and cancerous B-CLL cells using the three primary age classes as binary categorical features (Fig. 3C). Across all cell states, we observed that each age category was significantly assortative. Notably, the oldest UC gene category consistently exhibited high assortativity values, indicating a persistent clustering tendency of these genes throughout cell differentiation and malignant transformation. Interestingly, EM genes display high ChAs values in hESC and B-Cells, and decline significantly during oncogenesis. Conversely, MM genes, which initially have the lowest values in both hESC and B-Cells, show a marked increase in clustering in CLL cancer cells. Overall, these findings indicate a substantial reorganization of interactions among both EM and MM gene groups during oncogenesis: strong clustering of EM genes is partially disrupted in cancer, while MM genes are reorganized into much stronger clusters.

So far, ChAs analysis has provided insights into chromatin features associated with 3D genome contacts, here highlighting that genes of specific ages cluster together. To further understand which specific proteins might be implicated in mediating this spatial organization, we performed a variation of ChAs analysis, denoted as deltaChAs (ΔChAs). Briefly, this method measures the difference in ChAs values obtained in subnetworks of chromatin fragments with or without a specific feature to determine whether it significantly contributes to clustering a gene group together (ΔChAs > 0) or not (ΔChAs < 0) (see “Materials and methods” section for details).

We decided to test ΔChAs of several previously identified age-associated chromatin features in hESC, B-Cell, and B-CLL networks across each main age category (Fig. 3D). We observed positive ΔChAs for UC genes associated with Pol II in all three cell types, suggesting a role of Pol II in maintaining preferential contacts among UC genes in all cell states (c.f. Fig. 3C). The EM age class consistently exhibits positive ΔChAs z-scores for Polycomb targets compared to non-targets in all cell states, suggesting that the decrease of ChAs z-score for EM genes in B-CLL is primarily due to the disruption of preferential interactions among Polycomb-targeted EM genes, which are preserved in hESC and B-cells. Lastly, despite their low abundance and weaker clustering in hESC and B-cells, we observed positive ΔChAs for MM genes associated with Lamina in all three cell states. This underscores the potential importance and specificity of LAD in the clustering of genes within the MM age category across different cell states, which is especially strong in CLL cells.

Positive ΔChAs values of EM genes for Polycomb observed in all cellular states suggest that the clustering of EM genes could be mediated by Polycomb across differentiation. Other factors might be important in mediating the clustering and repression of developmental genes, specifically in stem cells. Despite the limited number of ChIP-seq datasets publicly available, we were able to calculate ΔChAs in hESC and B-cells for several TFs, showing that several of them might contribute to maintaining the clustering of UC genes and EM genes, specifically TBP, KMT2A (hESC), and STAT3 (B-Cell) for UC genes, and CTCF (hESC) for EM genes (Supplementary Fig. S3E).

To determine whether similar chromatin reorganization occurs during progression from healthy tissue to benign tumors and solid cancer, we analyzed data from a recent study on the transcriptome and 3D epigenome of CRC, covering three stages of progression: normal mucosa cells, benign polyps as an intermediate stage, and malignant adenocarcinoma cells [60]. We investigated gene expression patterns and ChAs scores in chromatin networks reconstructed for these stages, uncovering specific dynamic changes across stages (see Supplementary Fig. S3F–H, Supplementary Text S2 for description and Supplementary Text S3 for method).

In the context of 3D chromatin organization, our study reveals distinctive genome spatial patterns associated with gene evolutionary ages. Further analysis identified several chromatin features responsible for the 3D clustering of each specific evolutionary age. We observed that RNA Pol II facilitates preferential interactions among the oldest unicellular genes, aligning with their strong and stable expression, a trend consistent across various cell states. Lamina proteins could play a role in mediating interactions of mammalian-specific genes, showing moderate clustering in hESC and B-cell, and a notable increase in CLL. Polycomb proteins modulate the assortativity of EM genes, a phenomenon particularly prominent in both undifferentiated and differentiated cells compared to cancerous states. This suggests a significant reorganization of EM gene interactions driven by Polycomb activity alteration during oncogenesis.

## Discussion

### Investigating the link between gene age, regulation, and chromatin 3D organization

With the purpose of investigating potential mechanisms that relate genes’ evolutionary origins to their regulation and spatial organization, we interrogated several datasets. Our results underscore relationships between evolutionary gene ages with several expression and epigenomic features, as well as with chromatin structure. We expand on previous observations linking gene ages to the structure of topologically associating domains [61, 62, 9], finding gene age to be globally assortative in chromatin networks, with strong preferential clustering of genes of a particular age in specific cell states. Moreover, we suggest that the regulation of these different gene subsets could rely on different molecular mechanisms.

Given the known earlier replication timing of older housekeeping genes [8] as well as evidence of 3D organization of replication timing domains [63], of replication origin activation [58], and the assortativity of several chromatin states [64], these findings align with existing knowledge. Indeed, polycomb factors clearly associated with 3D structure have recently been implicated in origin activation [65], and spatial organization of genes according to function was suggested over a decade ago [66–69], while the importance of genes’ radial positioning is also well known [70, 71] and altered in cancer [72]. We here propose hypotheses on the factors establishing this organization and how it is modulated across differentiation and oncogenesis. Specifically, each gene age class exhibits distinct properties, which we will here summarize and consider in the context of relevant literature.

### Genes of unicellular origin: expression, regulation, and alterations in cancer

The oldest genes, of unicellular origin, UC, display higher expression levels, lower inter-individual EV, and feature active chromatin marks. Exploration of EV across individuals and tissues recently suggested that old genes would display low variability thanks to having broad promoters and multiple regulatory motifs [22]. It is well known that the oldest genes are involved in basic processes in the cells (housekeeping) [46, 73, 74]. These genes have the highest Pol II pausing indices, likely influencing their expression regulation. Interestingly, the importance of this mechanism tends to decrease during cell differentiation and increase during carcinogenesis. Previous studies demonstrated that the oldest (UC) genes tend to exhibit higher gene expression levels across various solid tumor samples [12] and cluster on protein–protein interaction networks [11]. Specifically, our findings suggest that in all cell types and states the oldest unicellular genes exhibit more pronounced connectivity among themselves, forming prominent clusters in the chromatin network. This was confirmed at all three stages of CRC investigated. Our results suggest that Pol II binding is involved in clustering of UC genes, while potentially maintaining coordination of their expression across differentiation and oncogenesis, in line with Pol II roles in chromatin organization [75, 76]. Despite our work focussing only on gene promoter regions, it has been recently shown that profiles of Pol II intergenic binding are sufficient to cluster samples by cell line, tissue, and cancer type [77], suggesting that coordination of Pol II bound genes could happen via complex networks of regulatory elements, as also suggested by analysis of Pol II binding on mESC chromatin networks [64]. Moreover, it has been shown that establishment of replication timing, which is closely related to 3D organisation in early development, is impacted by Pol II [78].

Overall, our findings suggest that oncogenesis may reinstigate a balance characterized by high expression of old genes typical of undifferentiated states, which could lead to expression of ancestral and developmentally early programmes producing a shift towards unicellular phenotypes, in line with the atavistic theory [79]. These phenotypes would include the Warburg effect [80] and alterations in cell–cell communication as the tissue homeostasis is disrupted [17].

### Metazoan genes and Polycomb repression

EM genes appeared with the evolution of multicellularity and complex body plans. They display intermediate expression and variability levels and enhanced Polycomb associated marks (H3K27me3). Polycomb protein complexes predominantly regulate genes that emerged during the metazoan period, aligning with the role of these genes in establishing body plans and their regulated suppression in early phases of development. Notably, the percentage of Polycomb targets seems to increase in differentiated cells and subsequently decrease in cancer, especially for EM genes. Those genes also demonstrate preferential interactions, but this pattern is lost in B-CLL cells, potentially in association with loss of cell identity. During development, Hox genes play a crucial role in forming the body plan [81, 82] and rely on Polycomb group proteins for regulation [52, 83, 84]. Moreover, numerous studies have demonstrated that Hox genes are implicated in cancer [85, 86]. Polycomb protein levels could modulate the assortativity of EM genes, a phenomenon particularly prominent in both undifferentiated and differentiated cells compared to cancerous states. The high value of ChAs z-score for EM genes in hESC aligns with earlier ChAs studies in mouse ESC [64].

This suggests a significant reorganization of EM gene interactions driven by loss of Polycomb activity during oncogenesis, which was recently confirmed to produce cancers in Drosophila in the absence of any genomic alteration [1, 16]. We cannot be sure of the relevance of these mechanisms in solid cancers, but our analysis of successive stages of CRC shows a similar picture, with an overall decrease in preferential interactions of EM genes compared to those of UC genes from healthy mucosa, to polyp to adenocarcinoma, with the biggest change happening already at the polyp stage.

### Mammalian-specific genes: DNA methylation and chromatin reorganization

Lastly, the newest mammalian specific genes (MM), displayed higher levels of DNA methylation and methylation variability in their promoters, potentially explaining their overall lower expression level. The relationship between DNA methylation at promoters and gene expression remains poorly understood, with recent reports that sequence variants might underlie the observed negative correlation [87]. However, the higher EV of new genes would be consistent with a less stable genome, accumulation of sequence variants, potentially through errors accumulated in late replication, and generally faster evolution [8]. Recent peripheral enhancer regions characterized by H3K9me2 only might also play a role in regulation of MM genes [88].

Evidence from yeast suggested a strong relation between variability of genes across single cells, across individuals, across species, and in response to external changes [89], consistent with the fact that highly variable and recent mammalian genes such as those related to immunity display faster genomic evolution [90, 91].

During hESC differentiation into cardiomyocytes, genes in the MM class are predominantly up-regulated, reflecting the development of a lineage-specific gene expression profile. These findings may also be attributed to the increased variability of gene expression observed across pluripotent cells; low-level expression of MM genes might be due to different cells expressing different genes in hESC. During differentiation, lineage-specific genes become upregulated in all or most cells, resulting in strong upregulation at the population level.

Interestingly, we found genes located at the nucleus periphery enriched in the MM class. The importance of radial positioning of genes in relation to their transcriptional state has been known for close to two decades [66, 69]. Given that genes interacting with nuclear lamina typically exhibit repressed expression [92, 93], this finding is consistent with the lower expression levels observed for the youngest genes (c.f. Fig. 1A). This association with the lamina might also in part explain the preferential interactions between MM genes, which is weak in stem and differentiated cells but becomes predominant in cancer, in which these genes are more strongly repressed as cell identity is lost. The effects of higher evolutionary plasticity of newer genes [8] might be controlled by their relegation to nuclear peripheral regions, where they are only expressed in very specific conditions such as cancer cells. It is also possible that rewiring of chromatin in cancer alters the regulatory mode of specific gene classes, linking chromatin structure to expression noise levels via control of burst size and frequency [91]. According to our results, the increase of MM genes’s preferential interactions during oncogenesis is less evident in CRC, which could either be due to the lesser quality of the chromatin networks reconstructed from this dataset, or to the larger heterogeneity of cells included in the cancer samples compared to isolated B cells. We expect a large proportion of cells in these samples to not be cancerous, including immune cells, fibroblasts, and other healthy cells, potentially diluting the signals that are more evident in CLL samples. These differences could also relate to specific characteristics of oncogenesis in blood malignancies and solid cancers. If solid cancer phenotypes can be related to a loss of cellular identity due to alterations of the tissue context, in leukemia the tissue context is totally absent (other than in the bone marrow and in lymph nodes). This could explain why the re-expression of UC genes is not observed in this cancer type (data not shown).

This work raises a series of hypotheses but we must mention several limitations of this study. First and foremost, we investigated published datasets integrating chromatin contact maps with epigenomic marks from different studies. These datasets may refer to cells in slightly different conditions and are likely influenced by biases specific to each type of experimental assay. Secondly, the ChAs measures depend, to a certain extent, on the size and characteristics of the contact networks being compared. To address this, we limited our comparisons to ChAs z-score values of gene age categories within a single chromatin network (e.g. hESC, B-cell, B-CLL) rather than comparing across different networks. Thirdly, not all genes are present in the promoter networks, due to experimental limitations, potentially affecting our results. As a fourth point, when studying chromatin structure of cancer cells, it is hard to guarantee that changes in chromatin conformation are not confounded with genome alterations. Additionally, we are here studying only the human genome 3D structure, and chromatin organization principles have changed across evolution [94, 95]. Moreover, single-cell specific chromatin organization cannot be captured by population-aggregated data, which only informs us on global trends and might be misleading [96].

Finally, another limitation is due to the lack of datasets describing promoter-centered chromatin structure in primary solid cancer cells, which prompted us to look at leukemic cells and one of the only examples available of 3D epigenomes of primary solid cancer samples. Differences between B-cells and B-CLL cells might be less pervasive than those between healthy cells in a tissue and their oncogenic counterparts.

## Conclusion

In conclusion, despite these limitations, the strong relationship we observed between gene age, EV, regulation, and 3D localization, combined with evidence that specific genes relocate within the nucleus during oncogenesis, prompts us to propose that the reshaping of chromatin during oncogenesis may modulate the variability and plasticity of phenotypes. Further work will have to be devoted to determining whether changes in the regulation of specific genes or a global rearrangement of genes of different ages in three-dimensional space contribute to the observed alterations in variability (across cells) and plasticity (over time) during differentiation and loss of cell identity, potentially sufficient to promote oncogenesis [97].

## Supplementary Material

gkaf084_Supplemental_Files

## Data Availability

No new data were produced for this study. The full list of previously published datasets used is provided in Supplementary Table S1. The script and processed datasets used for analysis and to generate main figures in this study are publicly available on Figshare at https://doi.org/10.6084/m9.figshare.26977654 and on GitHub at https://github.com/VeraPancaldiLab/Gene_Ages_3D_Paper.
