# Supplementary material for: Global chromatin reorganization and regulation of genes with specific evolutionary ages during differentiation and cancer
Source: Nucleic Acids Res. 2025 Feb 18;53(4):gkaf084. doi: 10.1093/nar/gkaf084 (PMC11833689; doi:10.1093/nar/gkaf084)
Supplement: gkaf084_Supplemental_Files [file gkaf084_supplemental_files.zip › Supplementary_Texts_and_Figures.pdf]

Supplementary Text and Figures

Supplementary Text S1

COSMIC genes enrichment analysis

To investigate whether genes involved in expression deregulation and regulatory mechanisms during cell differentiation and oncogenesis are more enriched in oncogenes or tumor suppressor genes (TSGs), we cross-referenced our results with the COSMIC 1st tier gene list of known oncogenes and TSGs. First, we examined the distribution of COSMIC oncogenes and TSGs across the three main gene age classes (**Supplementary Fig. 2D**). Approximately two-thirds of oncogenes belong to the EM class, one-third to the UC class, and very few to the MM class. In contrast, TSGs are almost equally distributed between the UC and EM classes, with very few in the MM class. Thus, oncogenes are slightly more prevalent in the EM class, while TSGs are more prominent in the UC class. Next, we generated heatmaps showing the enrichment of oncogenes and TSGs in up- or down-deregulated genes during cardiomyocyte differentiation, in genes targeted by polycomb proteins in hESCs, B-cells, and B-CLL, and in the top 25% of genes with the highest PolII pausing index across these three cell states (**Supplementary Fig. 2E**). The intersection of the Cardiomyocyte versus hESC differential analysis with the COSMIC gene list of oncogenes and TSGs revealed an enrichment of both oncogenes and TSGs among EM UP-regulated genes. Cross-referencing with polycomb protein targets indicated that the majority are oncogenes, with a smaller portion being TSGs. Additionally, analysis of the PolII pausing index revealed a marked enrichment of TSGs in paused UC genes, and of both oncogenes and TSGs in paused EM genes. To assess the statistical significance of these enrichments, we performed Fisher's exact tests (**Supplementary Fig. 2F**). These tests showed no significant enrichment of deregulated genes for either oncogenes or TSGs during cardiomyocyte differentiation. However, polycomb protein target analysis revealed significant enrichment of oncogenes among EM polycomb-targeted genes across hESCs, B-cells, and B-CLL. Additionally, PolII pausing index analysis indicated significant enrichment of TSGs in UC-paused genes across all three cell states.

In summary, these results suggest that a significant proportion of EM polycomb-targeted genes are oncogenes while many UC-paused genes are tumor suppressors and tend to maintain stable expression across different stages of differentiation and oncogenesis. While these analyses do not directly assess the impact of genetic alterations in these genes, which are typically at the origin of their classification as oncogenes or tumor suppressors,

mutations are likely to have limited impact on genes that are not actively expressed and may have a stronger effect when the gene is derepressed.

Supplementary Text S2

Colorectal cancer analysis

To investigate whether similar changes in chromatin organisation can be observed during the progression from a healthy tissue through a benign tumour stage towards a solid cancer, we took advantage of a recently published study characterising the transcriptome and 3D epigenome of several patient samples at successive stages of colorectal cancer (CRC), including healthy mucosa, benign polyp and adenocarcinoma (AdeCa) (3).

Firstly, we assessed differential gene expression analysis to investigate the regulation of oncogenes and tumor suppressor genes (TSGs) across the three stages (**Supplementary Fig. 3F**). We found 469 up- and 1197 down-regulated genes in polyp vs. mucosa, 1757 up- and 1197 down-regulated genes in AdeCa vs. polyp, and 1882 up- and 1795 down-regulated genes in AdeCa vs. mucosa. The oncogene *MET* is significantly up-regulated during the transition from mucosa to polyp (4). In the transition from mucosa to AdeCa, we observed the up-deregulation of additional oncogenes from the EM class with known involvement in CRC, including *HoxC11*, *SIX1*, and *TLX1* (5, 6). Additionally, *INSL5*, an EM-class TSG, is down-deregulated, consistent with previous reports (7).

Secondly, we analyzed changes in gene expression across the different stages and related them globally to gene ages (**Supplementary Fig. 3G**). Considering significantly differentially expressed genes between polyp and mucosa, we observed an overall down-regulation of EM and MM genes with a very slight up-regulation of UC genes. While genes from all three age classes are on average up-regulated between polyp and AdeCa stages, a direct comparison of AdeCa with mucosa revealed up-regulation of UC genes, with slight down-regulation of EM and MM genes. These findings suggest that Polycomb repression is still in place at the polyp stage, and is primarily lost during the transition to malignancy. MM genes are broadly down-regulated at the polyp stage, while they are up-regulated at the AdeCa stage. In contrast, UC genes show a consistent up-regulation across the three successive stages, potentially indicating their dual role in proliferation regulation, already happening at the polyp stage, and driving the loss of cellular identity, which is more closely associated with malignancy.

To study the alteration of 3D organisation of genes of different ages during this progression towards cancer, we measured gene age assortativity in the chromatin networks reconstructed from Hi-C datasets for each of the three stages of oncogenesis (**Supplementary Fig. 3H**). Our analysis revealed that UC genes consistently maintain high assortativity across the three stages of oncogenesis, consistent with our results on CLL. In

contrast, EM genes exhibit a relative decrease in assortativity compared to UC genes at both the polyp and Adenocarcinoma stages, while MM genes show a relative increase in assortativity in these stages. However, these tendencies are less pronounced than those observed in hESC, B-cell, and B-CLL comparisons.

Supplementary Text S3

Colorectal cancer analysis - *Method*

To study the relationship between gene age, gene expression, and chromatin structure in a solid cancer model, we used the dataset generated by Zhu and colleagues on colorectal cancer (CRC) (3). This dataset contains samples representing different stages of CRC progression, from normal mucosa to benign polyp as an intermediate stage and finally malignant adenocarcinoma (AdeCa). It includes 24 RNA-seq samples and 33 mHi-C samples across the three stages. For RNA-seq analysis, we used the count matrix obtained from the GEO database with accession number GSE207949. Differential gene expression analysis was performed using DESeq2 (v1.46.0), and comparing each pair of the three stages. Genes with an adjusted p-value ≤ 0.05 and log2 fold change ≥ 1 or ≤ -1 were considered significantly deregulated. The 3D chromatin structure analysis was performed starting from the Hi-C files (.hic) produced by Zhu *et al.* (3), under GEO accession number GSE207951. These files were converted from .hic to .cool format at a resolution of 50kb using the hicConvertFormat hic2cool function from the HiCExplorer utilities (v3.7.2) (8). Resulting .cool files were further normalized using the Knight-Ruiz (KR) method with the --correction_name parameter. Significant Hi-C interactions were called using Chromosight (v1.6.2) (9) with a threshold parameter of --pearson 0.2. Promoter-promoter interactions were defined as identified contacts where both fragments overlapped a gene TSS window from 200bp downstream to 2kb upstream. Chromatin networks for all samples within each stage (7 mucosa, 19 polyps, and 7 adenocarcinoma) were aggregated. ChAs and z-scores for each main age class were computed on these stage-specific chromatin networks as previously described.

Supplementary Figure 1

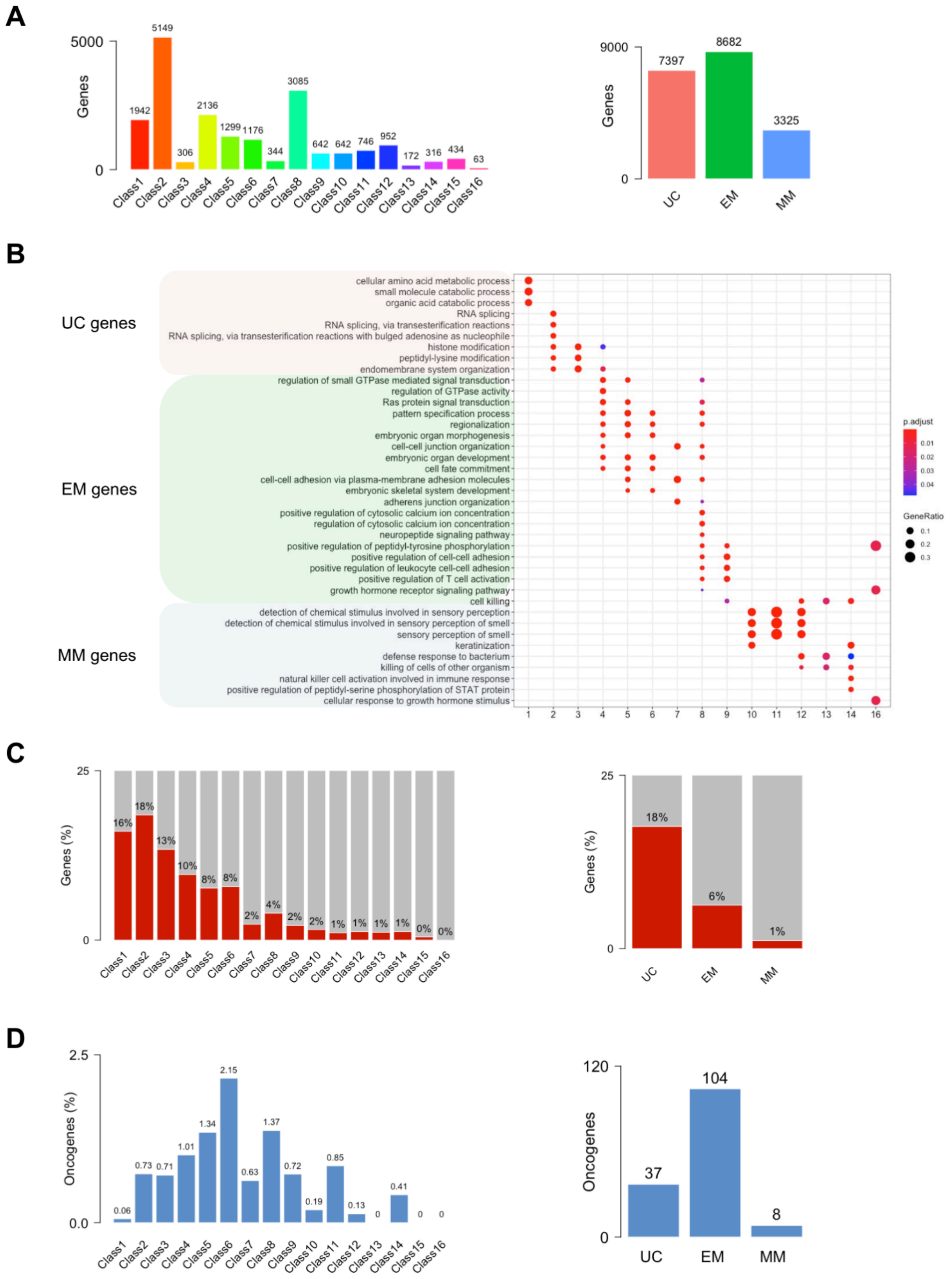

E

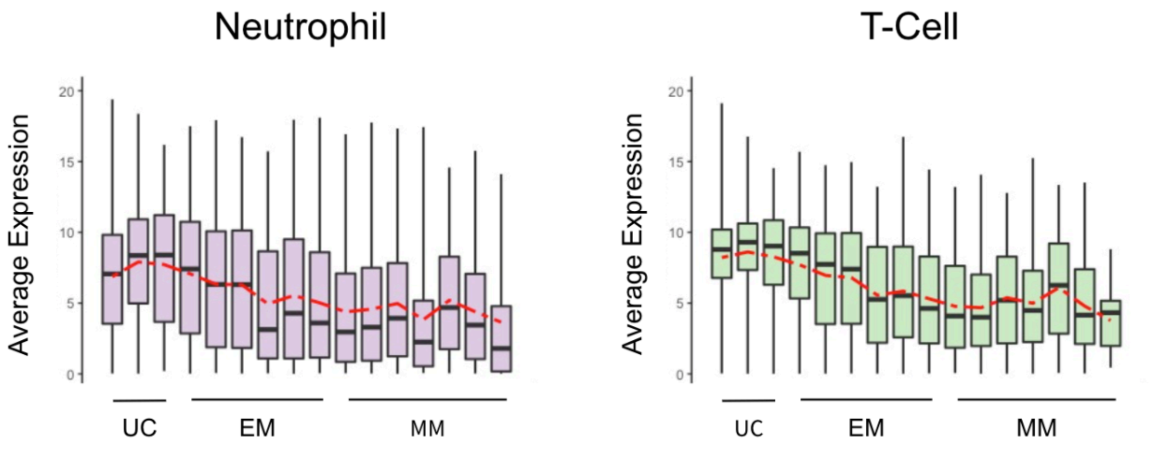
Neutrophil
T-Cell
Average Expression
20
15
10
5
0
UC
EM
MM

F

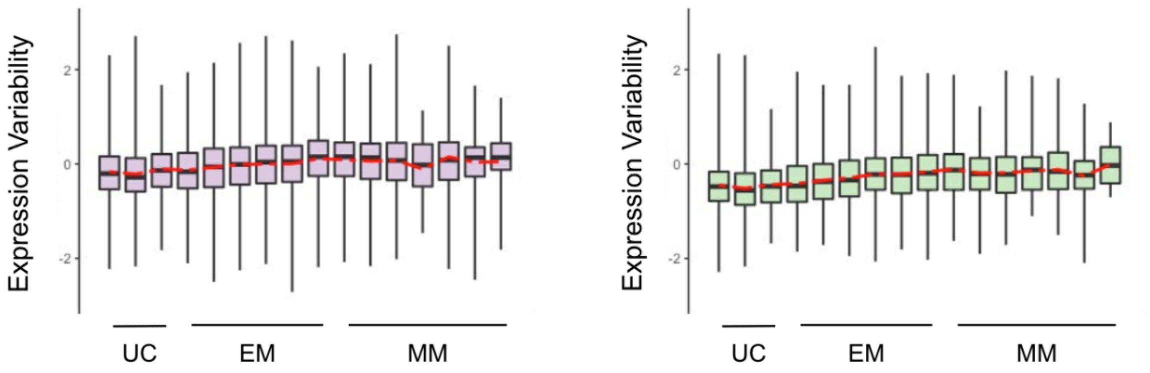
Expression Variability
2
0
-2
UC
EM
MM

G

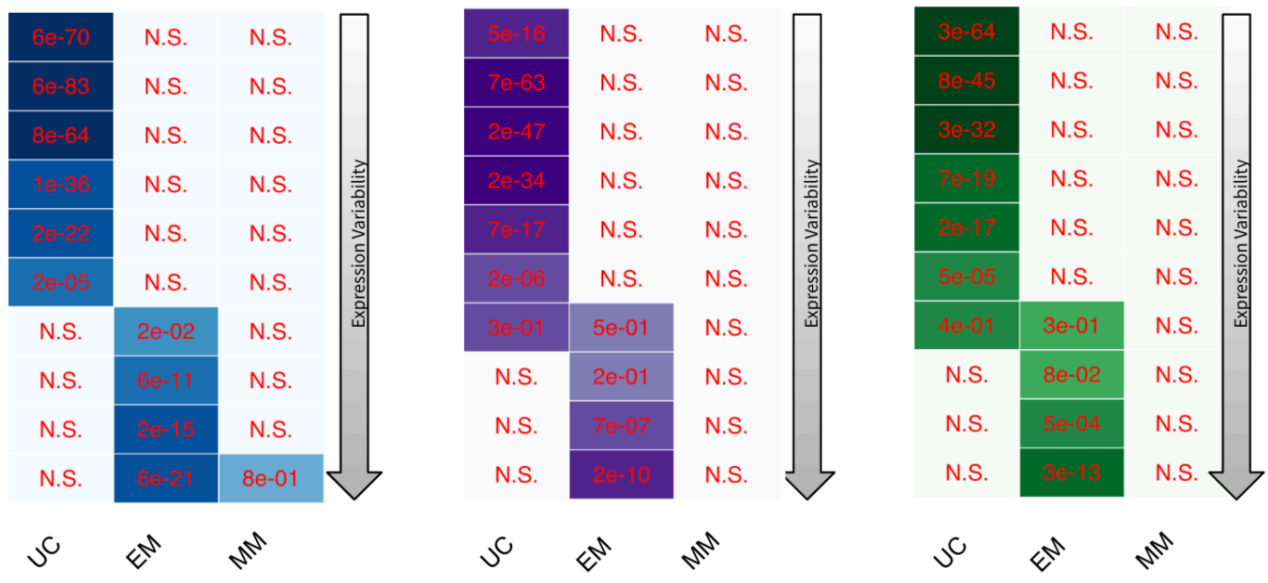
6e-70 N.S. N.S.
6e-83 N.S. N.S.
8e-64 N.S. N.S.
1e-36 N.S. N.S.
2e-22 N.S. N.S.
2e-05 N.S. N.S.
N.S. 2e-02 N.S.
N.S. 6e-11 N.S.
N.S. 2e-15 N.S.
N.S. 6e-21 8e-01
Expression Variability
5e-16 N.S. N.S.
7e-63 N.S. N.S.
2e-47 N.S. N.S.
2e-34 N.S. N.S.
7e-17 N.S. N.S.
2e-06 N.S. N.S.
3e-01 5e-01 N.S.
N.S. 2e-01 N.S.
N.S. 7e-07 N.S.
N.S. 2e-10 N.S.
Expression Variability
3e-64 N.S. N.S.
8e-45 N.S. N.S.
3e-32 N.S. N.S.
7e-19 N.S. N.S.
2e-17 N.S. N.S.
5e-05 N.S. N.S.
4e-01 3e-01 N.S.
N.S. 8e-02 N.S.
N.S. 5e-04 N.S.
N.S. 3e-13 N.S.
Expression Variability
UC
EM
MM

H

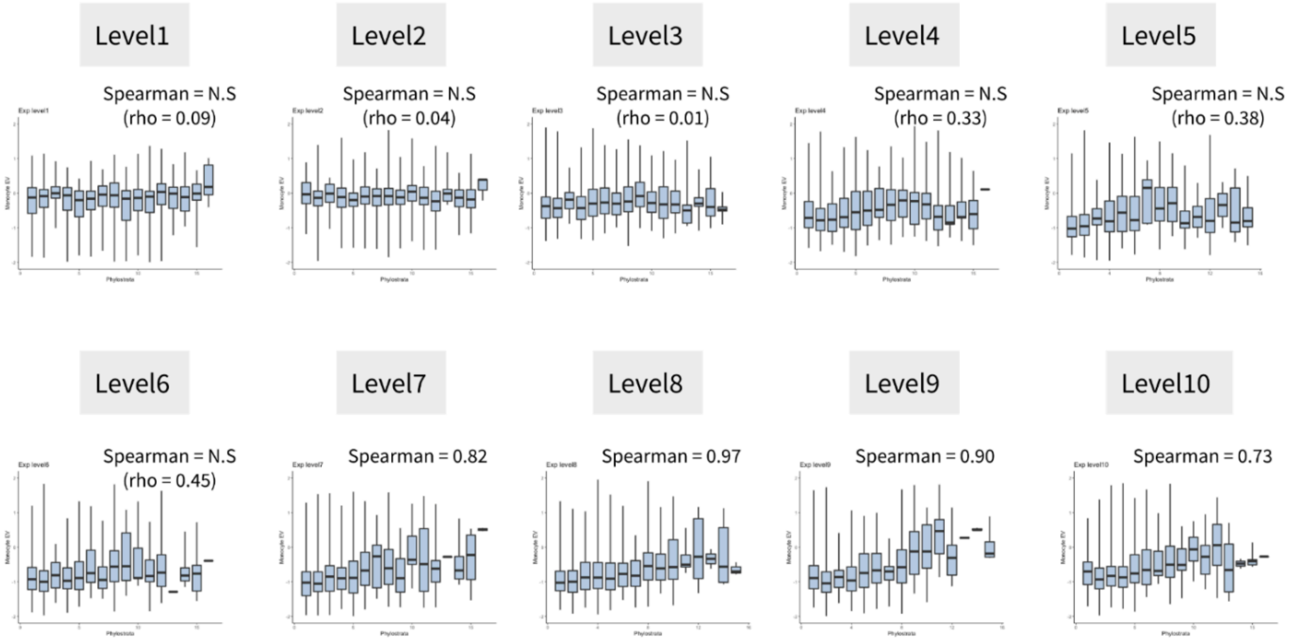
Level1
Level2
Level3
Level4
Level5
Spearman = N.S
(rho = 0.09)
Spearman = N.S
(rho = 0.04)
Spearman = N.S
(rho = 0.01)
Spearman = N.S
(rho = 0.33)
Spearman = N.S
(rho = 0.38)
Level6
Level7
Level8
Level9
Level10
Spearman = N.S
(rho = 0.45)
Spearman = 0.82
Spearman = 0.97
Spearman = 0.90
Spearman = 0.73

I

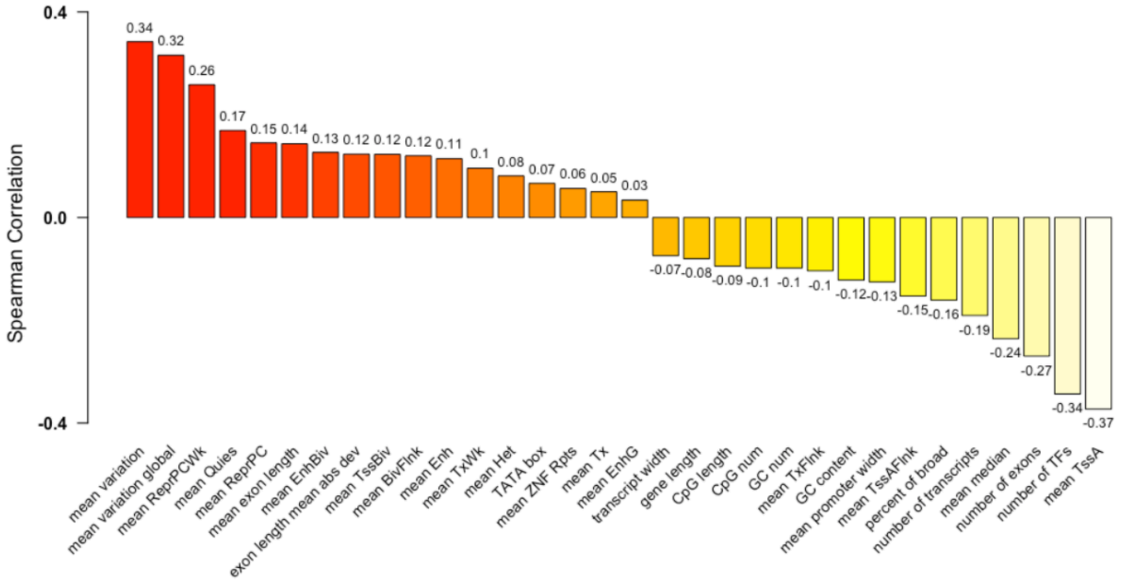
Spearman Correlation
0.4
0.0
-0.4
0.34
0.32
0.26
0.17
0.15
0.14
0.13
0.12
0.12
0.12
0.11
0.1
0.08
0.07
0.06
0.05
0.03
-0.07
-0.08
-0.09
-0.1
-0.1
-0.1
-0.12
-0.13
-0.15
-0.16
-0.19
-0.24
-0.27
-0.34
-0.37
mean variation
mean variation global
mean ReprPCWk
mean Quies
mean ReprPC
mean exon length
mean EnhBiv
exon length mean abs dev
mean TssBiv
mean BivFlnk
mean Enh
mean TxWk
mean Het
TATA box
mean ZNF Rpts
mean Tx
mean EnhG
transcript width
gene length
CpG length
CpG num
GC num
mean TxFlnk
GC content
mean promoter width
mean TssAFlnk
percent of broad
number of transcripts
mean median
number of exons
number of TFs
mean TssA

J

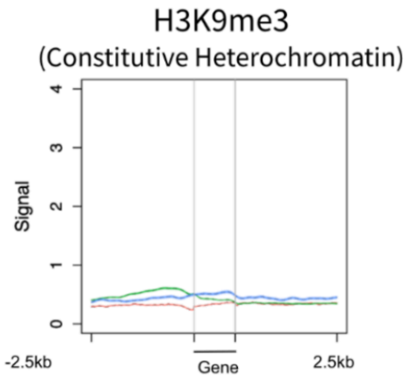
H3K9me3
(Constitutive Heterochromatin)
Signal
0
1
2
3
4
-2.5kb
Gene
2.5kb

K

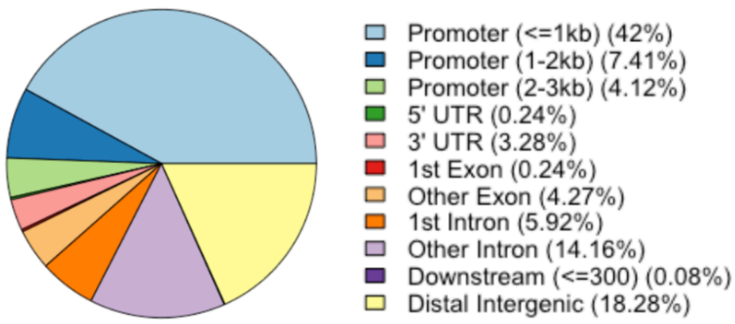
Promoter (<=1kb) (42%)
Promoter (1-2kb) (7.41%)
Promoter (2-3kb) (4.12%)
5' UTR (0.24%)
3' UTR (3.28%)
1st Exon (0.24%)
Other Exon (4.27%)
1st Intron (5.92%)
Other Intron (14.16%)
Downstream (<=300) (0.08%)
Distal Intergenic (18.28%)

Supplementary Fig1. (related to Fig.1)

A) Number of genes in each phylostratum (left) and in each main age class (right). **B**) Gene Ontology enrichment of genes from each phylostratum. **C**) Proportion of housekeeping genes in each phylostratum (left) and in each main age class (right). **D**) Proportion of Leukemia proto-oncogenes in each phylostratum (left) and in each main age class (right). Dataset from Jang *et al.*, 2015 (1). **E**) Boxplot of average inter-individual gene expression levels in Neutrophil (left) and T-Cell (right) across 16 gene age classes (Spearman test in neutrophil, Rho = -0.88, *p*-value < 2.2e-16. Spearman test in t-cell, Rho -0.85, *p*-value = 1.023e-05). **F**) Boxplots of inter-individual expression variability in Neutrophil (left) and T-Cell (right) across 16 gene age classes (Spearman test in neutrophil, Rho = 0.74, *p*-value = 0.001703. Spearman test in t-cell, Rho = 0.84, *p*-value = 1.932e-05). **G**) Fisher's Exact Tests of 10 EV decile levels and 3 main gene ages in Monocyte, Neutrophil and T-Cell, from left to right. Color shades represent the Jaccard index statistic (calculated as the ratio of intersection over union of sets), with lighter tones indicating lower values and darker tones indicating higher values. This confirms the link between EV and gene evolutionary ages. **H**) Boxplots of inter-individual expression variability in Monocyte across the 16 phylostrata for 10 gene expression variability level classes. **I**) Spearman correlations of various DNA variability properties with 16 gene age classes. **J**) Average H3K9me3 profile in Monocyte around the 3 main gene age classes (Red Unicellular genes, Green Early metazoan genes, Blue Mammal Specific genes) (2). **K**) Genomic localisations of all detected CpG islands in monocytes.

Supplementary Figure 2

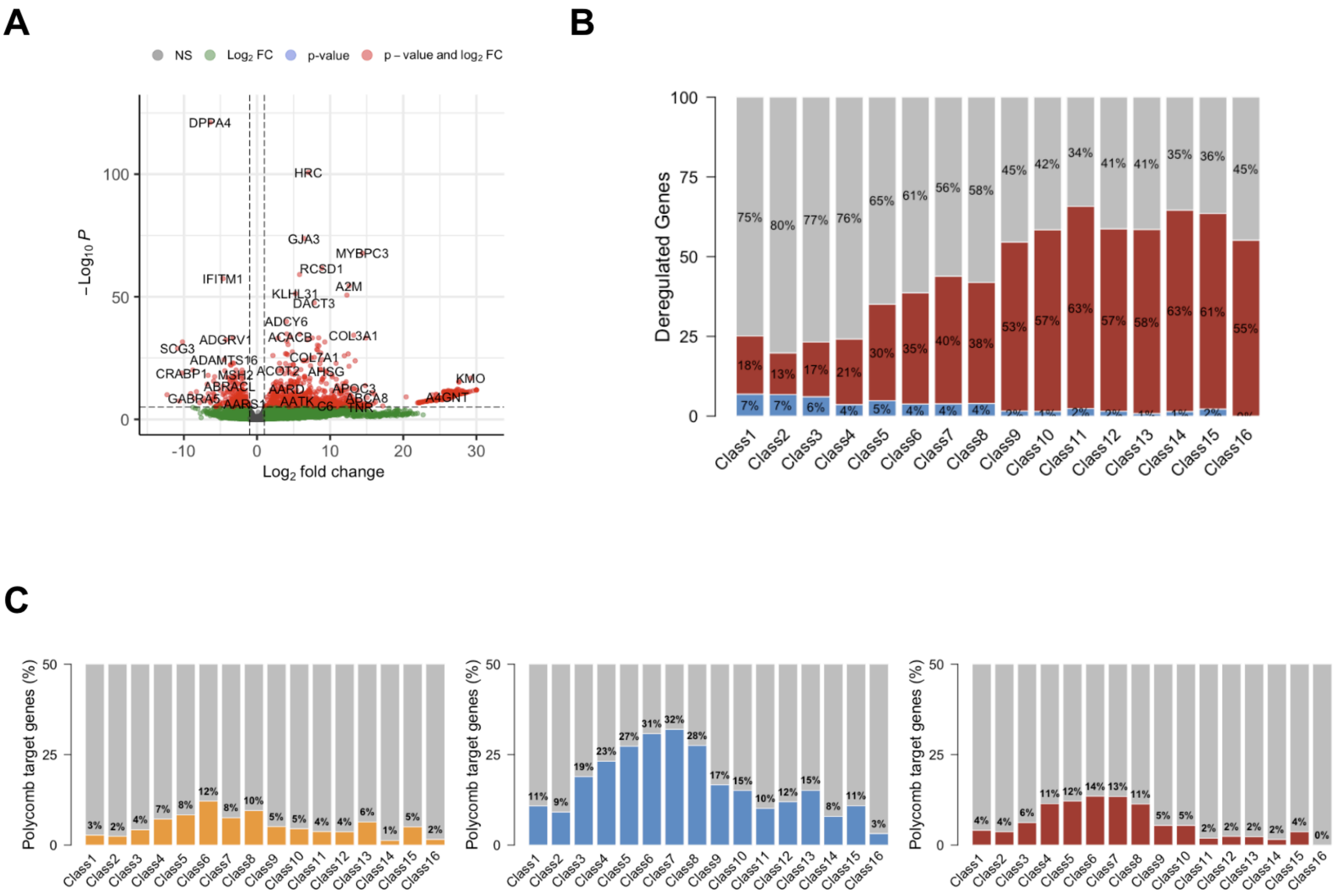

D

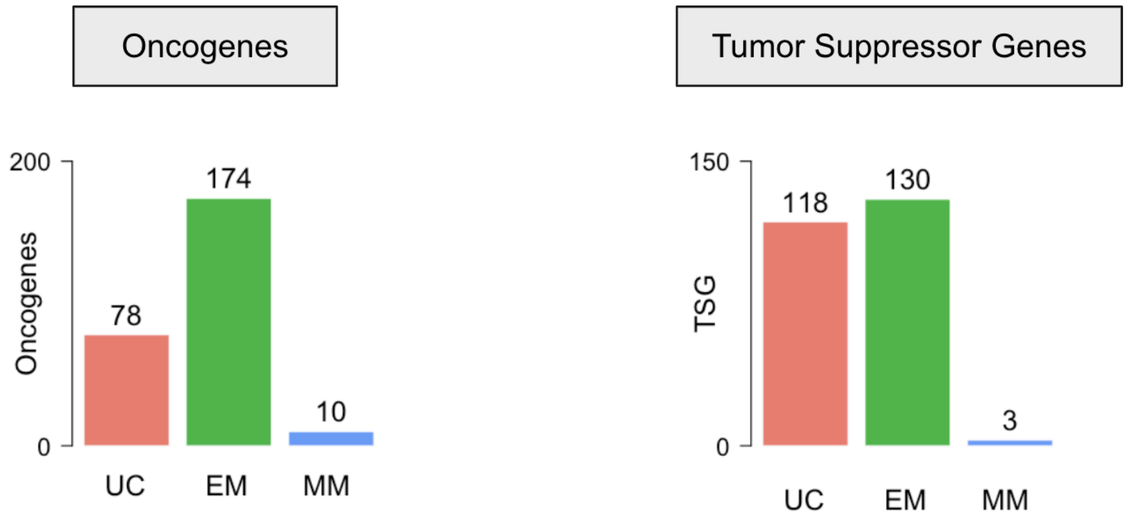
Oncogenes
200
0
Oncogenes
78
174
10
UC
EM
MM
Tumor Suppressor Genes
150
0
TSG
118
130
3
UC
EM
MM

E

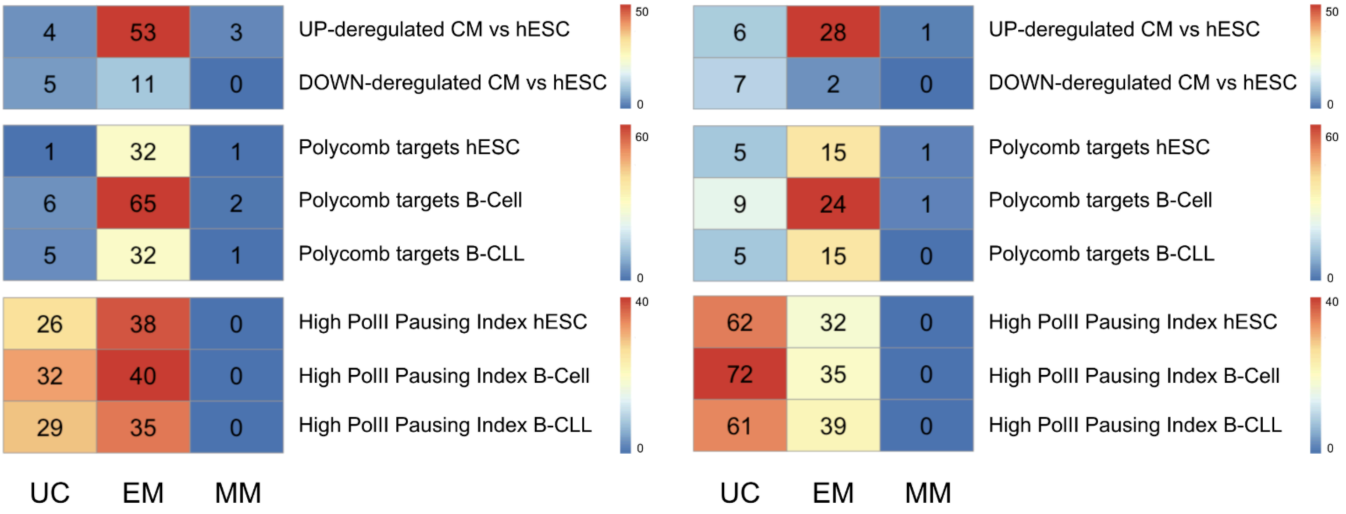
4 53 3 UP-deregulated CM vs hESC
5 11 0 DOWN-deregulated CM vs hESC
1 32 1 Polycomb targets hESC
6 65 2 Polycomb targets B-Cell
5 32 1 Polycomb targets B-CLL
26 38 0 High PolII Pausing Index hESC
32 40 0 High PolII Pausing Index B-Cell
29 35 0 High PolII Pausing Index B-CLL
UC EM MM
50 0
60 0
40 0
6 28 1 UP-deregulated CM vs hESC
7 2 0 DOWN-deregulated CM vs hESC
5 15 1 Polycomb targets hESC
9 24 1 Polycomb targets B-Cell
5 15 0 Polycomb targets B-CLL
62 32 0 High PolII Pausing Index hESC
72 35 0 High PolII Pausing Index B-Cell
61 39 0 High PolII Pausing Index B-CLL
UC EM MM

F

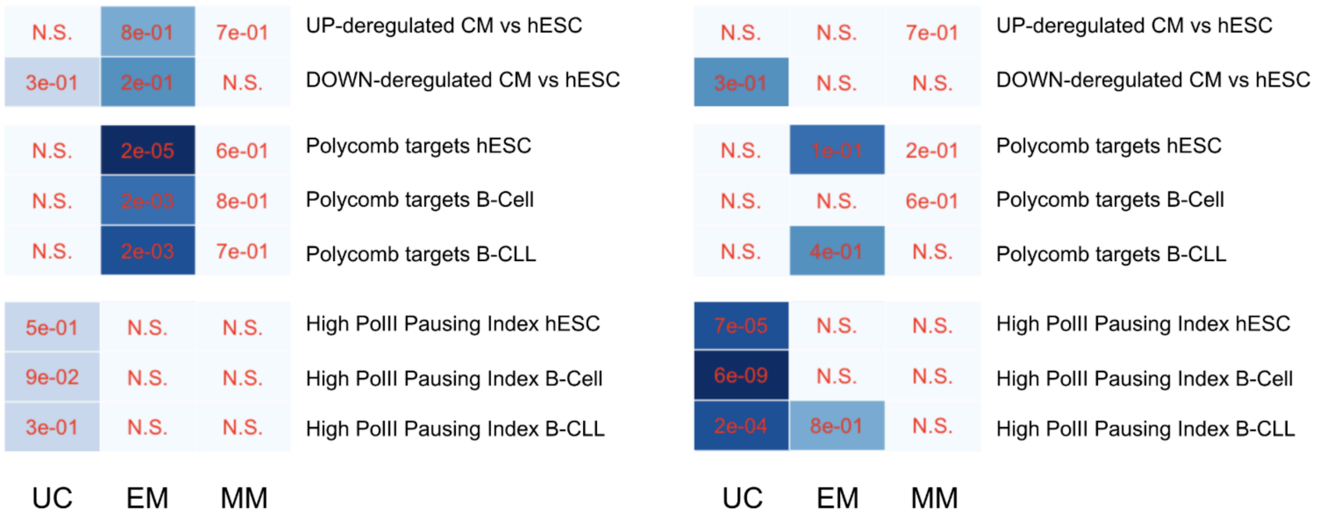
N.S. 8e-01 7e-01 UP-deregulated CM vs hESC
3e-01 2e-01 N.S. DOWN-deregulated CM vs hESC
N.S. 2e-05 6e-01 Polycomb targets hESC
N.S. 2e-03 8e-01 Polycomb targets B-Cell
N.S. 2e-03 7e-01 Polycomb targets B-CLL
5e-01 N.S. N.S. High PolII Pausing Index hESC
9e-02 N.S. N.S. High PolII Pausing Index B-Cell
3e-01 N.S. N.S. High PolII Pausing Index B-CLL
UC EM MM
N.S. N.S. 7e-01 UP-deregulated CM vs hESC
3e-01 N.S. N.S. DOWN-deregulated CM vs hESC
N.S. 1e-01 2e-01 Polycomb targets hESC
N.S. N.S. 6e-01 Polycomb targets B-Cell
N.S. 4e-01 N.S. Polycomb targets B-CLL
7e-05 N.S. N.S. High PolII Pausing Index hESC
6e-09 N.S. N.S. High PolII Pausing Index B-Cell
2e-04 8e-01 N.S. High PolII Pausing Index B-CLL
UC EM MM

Supplementary Fig2. (related to Fig.2)

A) Volcano plot of differentially expressed genes in Cardiomyocyte compared to hESC. **B**) Proportion of Down (blue), Up (red) and non (grey) deregulated genes in Cardiomyocyte compared to hESC across the 16 gene age categories. **C**) Proportion of Polycomb target genes across the 16 gene age categories in hESC (left), B-cell (middle) and CLL (right). **D**) Number of oncogenes (left) and tumor suppressor genes (right) classified in COSMIC first tier across each main age class. **E**) Heatmaps with number of COSMIC oncogenes (left) and tumor suppressor genes (right) in each gene set. **F**) Heatmap of p-values of enrichment of COSMIC oncogenes (left) and tumor suppressor genes (right) in each gene set. Color shades represent the Jaccard index statistic (calculated as the ratio of intersection over union of sets), with lighter tones indicating lower values and darker tones indicating higher values.

Supplementary Figure 3

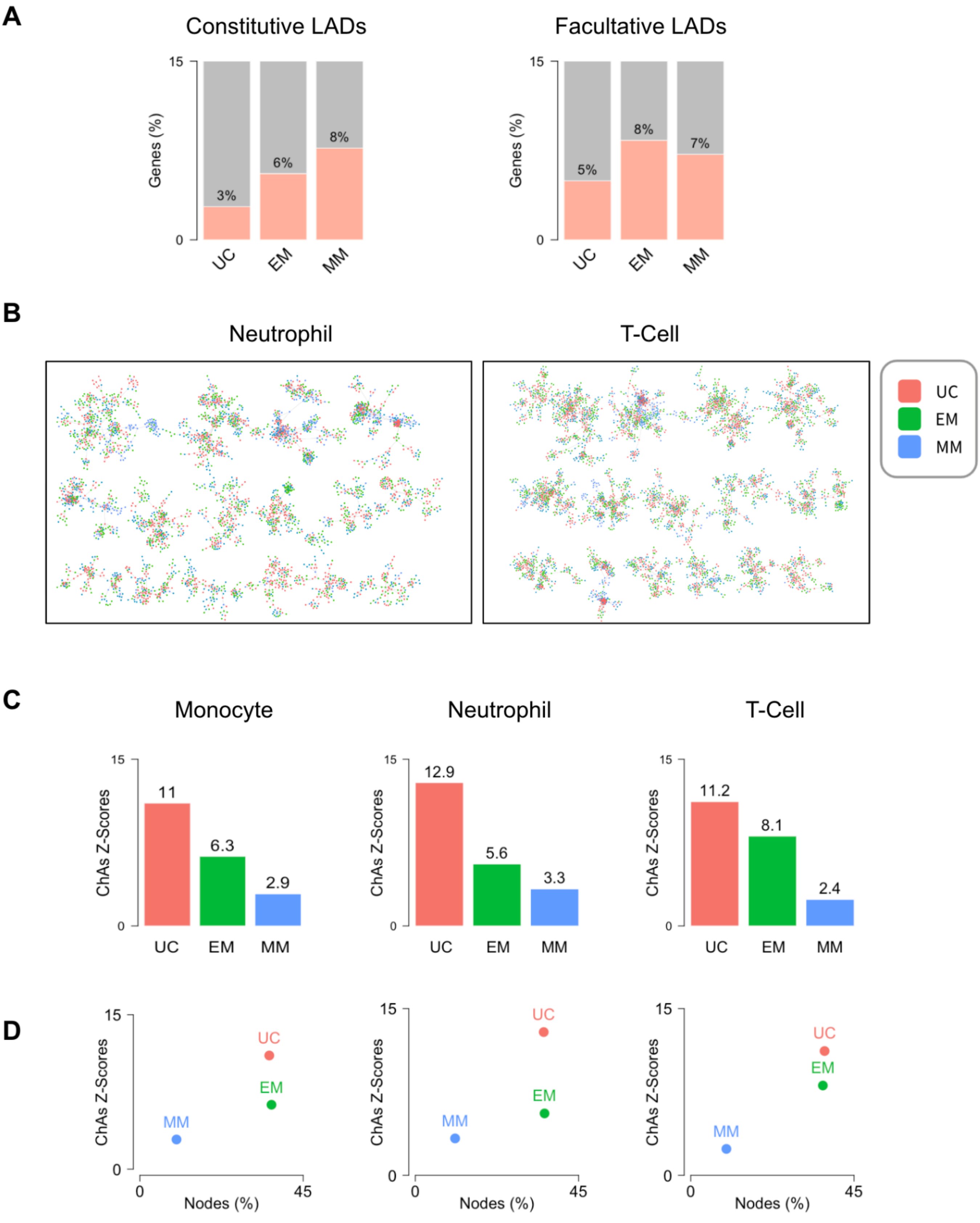

E

hESC

UC	EM	MM	
12.99	-3.62	-4.20	TBP
7.64	-1.85	-5.84	KMT2A
5.80	-0.43	-10.52	TP53BP1
5.80	-0.57	-7.87	EP300
3.67	0.29	-7.49	RNF2
4.98	-7.30	-3.68	GTF3C1
4.89	-3.68	-7.88	NR5A1
2.27	-3.05	-5.47	ZNF143
-6.44	-0.06	-3.85	RAD21
-6.07	-2.58	-2.59	ARID1A
-4.74	-2.75	-2.58	TFAP2A
-2.41	2.09	-3.41	CTCF
-4.17	-1.34	-5.19	Epitope
-3.40	-2.61	-3.44	NANOG
-1.60	-2.57	-4.74	RUNX1T1
-8.01	-10.31	-8.96	LEO1
-8.71	-9.85	-3.88	CCNE1
-9.15	-7.84	-3.98	LDB1
-7.68	-5.88	-0.87	SMAD3
-7.73	-5.27	-2.74	Biotin
-6.83	-6.24	-2.38	HSF1
-8.57	-5.60	-4.87	G-quadruplex
-9.31	-5.89	-3.79	TCF7L1
-7.38	-5.92	-3.88	NUDT21
-7.14	-5.84	-4.50	POU5F1
-8.35	-6.71	-3.99	KDM6A
-7.55	-6.98	-3.66	PAX7
-7.35	-7.12	-3.55	TP53
-5.51	-6.71	-2.52	GTF3C2
-5.25	-7.53	-4.92	SMAD2
-5.72	-5.99	-4.59	TP63
-5.58	-6.07	-3.75	RUNX1
-6.06	-6.29	-4.11	ZNF398
-6.26	-4.66	-4.69	GRHL2
-5.73	-4.48	-5.30	SOX2
-6.54	-6.73	-4.87	FOXH1
-7.39	-6.17	-5.34	BRF1
-7.07	-6.51	-6.27	LMO2
-1.38	-7.17	-4.00	BRD4
-2.97	-4.72	-5.27	FOXP1
-4.81	-5.02	-3.35	GATA3
-4.81	-4.58	-3.70	SMARCA4
-2.96	-4.65	-3.61	ZNF207
-3.70	-5.60	-3.17	KLF4
-3.27	-5.58	-4.09	NIPBL
-3.86	-5.81	-4.26	SMAD4

B-Cell

UC	EM	MM	
8.55	-6.17	-4.52	STAT3
3.52	-4.60	-5.07	BCOR
3.96	-4.56	-3.83	POU2AF1
3.07	-7.86	-3.96	POU2F2
3.49	-5.82	-2.80	BCL6
1.49	-5.71	-3.27	GFP
-1.34	-8.91	-4.96	FOXP1
0.41	-8.96	-4.07	RBPJ
-4.07	-7.33	-5.08	BACH2
-3.14	-7.71	-4.51	CREBBP
-2.50	-9.19	-3.37	MEF2B
-4.09	-9.01	-3.33	SMARCA4
-8.53	-9.87	0.56	Epitope
-4.21	-3.89	-3.99	CTCF
-7.20	-8.63	-4.81	EBNA1
-5.55	-9.27	-1.99	ZNF341
-5.63	-6.29	-4.03	EBF1
-5.60	-6.97	-2.51	CD74
-6.19	-6.79	-2.10	SPI1
-6.66	-5.98	-1.58	KMT2D
-7.29	-6.30	-0.89	NCOR2
-8.25	-7.16	-3.35	IRF4
-7.93	-7.94	-1.83	MAU2
-7.16	-8.28	-1.71	FOXO1
-7.25	-7.82	-2.40	RAD21

B-CLL

UC	EM	MM	
-3.35	0.24	-6.95	BRD4

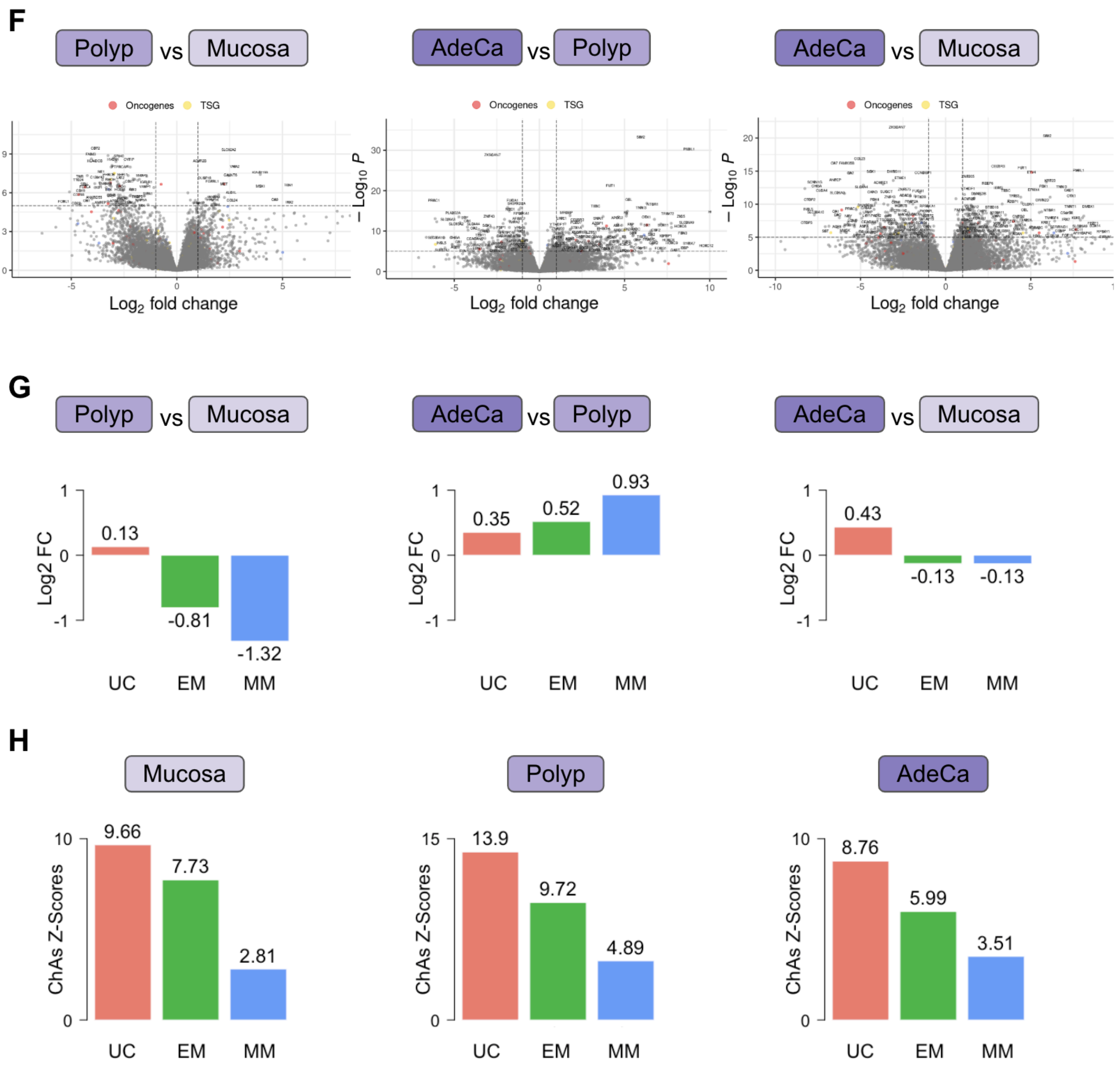
F
Polyp vs Mucosa
AdeCa vs Polyp
AdeCa vs Mucosa
Oncogenes
TSG
$-\log_{10} P$
$\log_2$ fold change
G
Polyp vs Mucosa
0.13
-0.81
-1.32
AdeCa vs Polyp
0.35
0.52
0.93
AdeCa vs Mucosa
0.43
-0.13
-0.13
Log2 FC
UC
EM
MM
H
Mucosa
9.66
7.73
2.81
Polyp
13.9
9.72
4.89
AdeCa
8.76
5.99
3.51
ChAs Z-Scores
UC
EM
MM

Supplementary Fig3. (related to Fig.3)

A) Overlaps with Lamina Associated Domains (LADs) annotated in multiple cell lines nd for genes belonging to the different age classes. **B**) Visualizations of the 3D promoter-promoter interaction networks in neutrophil (left) and T-cell (right) with node color representing gene age classes (red = UC, green = EM, blue = MM). **C**) ChAs z-scores compared amongst the 3 age classes in monocyte, neutrophil and T-cell promoter-promoter interaction networks. **D**) ChAs z-scores and related feature abundance of the 3 age classes in monocyte, neutrophil and T-cell promoter-promoter interaction networks. **E**) ΔChAs z-scores matrix of several transcription factors and chromatin features in hESC, B-Cell, and B-CLL. Colors represent ΔChAs > 0 in cyan and ΔChAs < 0 in red. **F**) Volcano plots of differentially expressed genes between successive stages of colorectal cancer (healthy mucosa, benign polyp and adenocarcinoma) from Zhu *et al*. (3). Oncogenes and tumor suppressor genes are highlighted in red and yellow, respectively. **G**) Comparison of average gene expression fold-change between successive stages of colorectal cancer (red = UC, green = EM, blue = MM, only genes with adjusted p-value ≤ 0.05 and log2 fold change ≥ 1 or ≤ -1 were considered. **H**) Comparison of gene age ChAs z-scores across the three age classes between mucosa, polyp, and adenocarcinoma, based on Hi-C-derived promoter-promoter interaction networks (**see Supplementary Text 2**).

SI References

1. Jang,S.H., Lee,S. and Chung,H.Y. (2015) Characterization of Leukemia-Inducing Genes Using a Proto-Oncogene/Homeobox Gene Retroviral Human cDNA Library in a Mouse In Vivo Model. *PLOS ONE*, **10**, e0143240.
2. Sigalova,O.M., Shaeiri,A., Forneris,M., Furlong,E.E. and Zaugg,J.B. (2020) Predictive features of gene expression variation reveal mechanistic link with differential expression. *Mol. Syst. Biol.*, **16**, e9539.
3. Zhu,Y., Lee,H., White,S., Weimer,A.K., Monte,E., Horning,A., Nevins,S.A., Esplin,E.D., Paul,K., Krieger,G., *et al.* (2024) Global loss of promoter–enhancer connectivity and rebalancing of gene expression during early colorectal cancer carcinogenesis. *Nat. Cancer*, **5**, 1697–1712.
4. Neklason,D.W., Done,M.W., Sargent,N.R., Schwartz,A.G., Anton-Culver,H., Griffin,C.A., Ahnen,D.J., Schildkraut,J.M., Tomlinson,G.E., Strong,L.C., *et al.* (2011) Activating mutation in MET oncogene in familial colorectal cancer. *BMC Cancer*, **11**, 424.
5. Xu,H., Zhang,Y., Peña,M.M., Pirisi,L. and Creek,K.E. (2017) Six1 promotes colorectal cancer growth and metastasis by stimulating angiogenesis and recruiting tumor-associated macrophages. *Carcinogenesis*, **38**, 281–292.
6. Chu,S., Ren,X., Cao,L., Ma,C. and Wang,K. (2024) HOXC11-mediated regulation of mitochondrial function modulates chemoresistance in colorectal cancer. *BMC Cancer*, **24**, 921.
7. Yang,X., Wei,W., Tan,S., Guo,L., Qiao,S., Yao,B. and Wang,Z. (2021) Identification and verification of HCAR3 and INSL5 as new potential therapeutic targets of colorectal cancer. *World J. Surg. Oncol.*, **19**, 248.
8. Wolff,J., Rabbani,L., Gilsbach,R., Richard,G., Manke,T., Backofen,R. and Grüning,B.A. (2020) Galaxy HiCExplorer 3: a web server for reproducible Hi-C, capture Hi-C and single-cell Hi-C data analysis, quality control and visualization. *Nucleic Acids Res.*, **48**, W177–W184.
9. Matthey-Doret,C., Baudry,L., Breuer,A., Montagne,R., Guiglielmoni,N., Scolari,V., Jean,E., Campeas,A., Chanut,P.H., Oriol,E., *et al.* (2020) Computer vision for pattern detection in chromosome contact maps. *Nat. Commun.*, **11**, 5795.
